# Measuring Health Information Dissemination and Identifying Target Interest Communities on Twitter: Methods Development and Case Study of the @SafetyMD Network

**DOI:** 10.2196/resprot.4203

**Published:** 2016-05-05

**Authors:** Venk Kandadai, Haodong Yang, Ling Jiang, Christopher C Yang, Linda Fleisher, Flaura Koplin Winston

**Affiliations:** ^1^ Center for Injury Research and Prevention Children's Hospital of Philadelphia Philadelphia, PA United States; ^2^ College of Computing and Informatics Drexel University Philadelphia, PA United States; ^3^ Perelman School of Medicine Department of Pediatrics University of Pennsylvania Philadelphia, PA United States

**Keywords:** Twitter, health information, dissemination, health communication, digital health

## Abstract

**Background:**

Little is known about the ability of individual stakeholder groups to achieve health information dissemination goals through Twitter.

**Objective:**

This study aimed to develop and apply methods for the systematic evaluation and optimization of health information dissemination by stakeholders through Twitter.

**Methods:**

Tweet content from 1790 followers of @SafetyMD (July-November 2012) was examined. User emphasis, a new indicator of Twitter information dissemination, was defined and applied to retweets across two levels of retweeters originating from @SafetyMD. User interest clusters were identified based on principal component analysis (PCA) and hierarchical cluster analysis (HCA) of a random sample of 170 followers.

**Results:**

User emphasis of keywords remained across levels but decreased by 9.5 percentage points. PCA and HCA identified 12 statistically unique clusters of followers within the @SafetyMD Twitter network.

**Conclusions:**

This study is one of the first to develop methods for use by stakeholders to evaluate and optimize their use of Twitter to disseminate health information. Our new methods provide preliminary evidence that individual stakeholders can evaluate the effectiveness of health information dissemination and create content-specific clusters for more specific targeted messaging.

## Introduction

The Centers for Disease Control and Prevention (CDC) uses Twitter as its sole microblogging platform, actively encouraging its use to reach stakeholders with relevant health information [1] and to provide a framework for health information dissemination best practices [2]. Web 2.0 and social media platforms like Twitter provide an opportunity to bridge the gap between innovation and dissemination by leveraging the viral spread of information across large networks of potential stakeholders. However, little is known about the ability of individual stakeholder groups to achieve their health information dissemination goals through Twitter.

Twitter allows for easy communication and spread of information through networks. Methods to determine the effectiveness of this communication are well documented and widely accessible through an application programming interface (API). Twitter information spread begins when a user posts a short message (currently limited to 140 characters) referred to as a tweet. Users (the stakeholders to which the CDC refers), including influential users, can spread health information through Twitter by encouraging others to follow their tweets. In addition to receiving the information, followers can spread the information to their followers through retweeting (ie, echoing a message by another user). This is the benefit of social media networks: important information can be spread rapidly by sending a message on Twitter to a large and active following base.

An analysis of data from the Health Information and National Trends Survey (HINTS) revealed 23% of Internet users actively engaged in social networking sites [3], and a 2013 study showed 74% of online adults use social networking sites, suggesting a more than 2-fold growth of social network users in the past 5 years [4]. In particular, 35% of Internet users who use Twitter are young adults (aged 18-29 years) or elderly (65 years and older), and nearly 30% are from racial and ethnic minority populations [5]. This is the second potential benefit of Twitter: special groups that might be difficult to reach through other channels can be reached on Twitter, where they are already actively engaged.

There is little information, however, about the effectiveness of Twitter in spreading health information disseminated by credible, nongovernmental individual or group stakeholders. Such individuals and groups are usually part of a network of professionals with whom they work closely, and the network is a crucial channel to spread credible materials in the field. Exploring the effectiveness of the information dissemination in such networks is significant for enhancing the promotion of valuable news and findings. So far, few techniques are available for optimizing dissemination of credible health information by linking user interests with content. In an effort to develop tools to improve this dissemination, the authors report on a methodology examining a case study of an existing Twitter network, @SafetyMD. This study aimed to develop and apply methods of (1) measuring the continued emphasis of health information themes as they spread through two levels of followers of @SafetyMD and (2) identifying targeted interest groups among the followers of @SafetyMD. The goal of this study was to advance methodology for the systematic evaluation and optimization of health information dissemination by stakeholders through Twitter.

## Methods

### The @SafetyMD Network

The Center for Injury Research and Prevention (CIRP) at the Children’s Hospital of Philadelphia has a research-to-action-to-impact outreach strategy that relies on a large network of child and adolescent professionals interested in injury prevention and treatment. Outreach professionals within the center work closely with scientists and engineers to translate evidence into credible messages and materials delivered mostly on the Web. One of the channels for disseminating this information is the official CIRP Twitter handle, @SafetyMD, launched in 2011 and led by the center’s scientific director.

**Figure 1:**
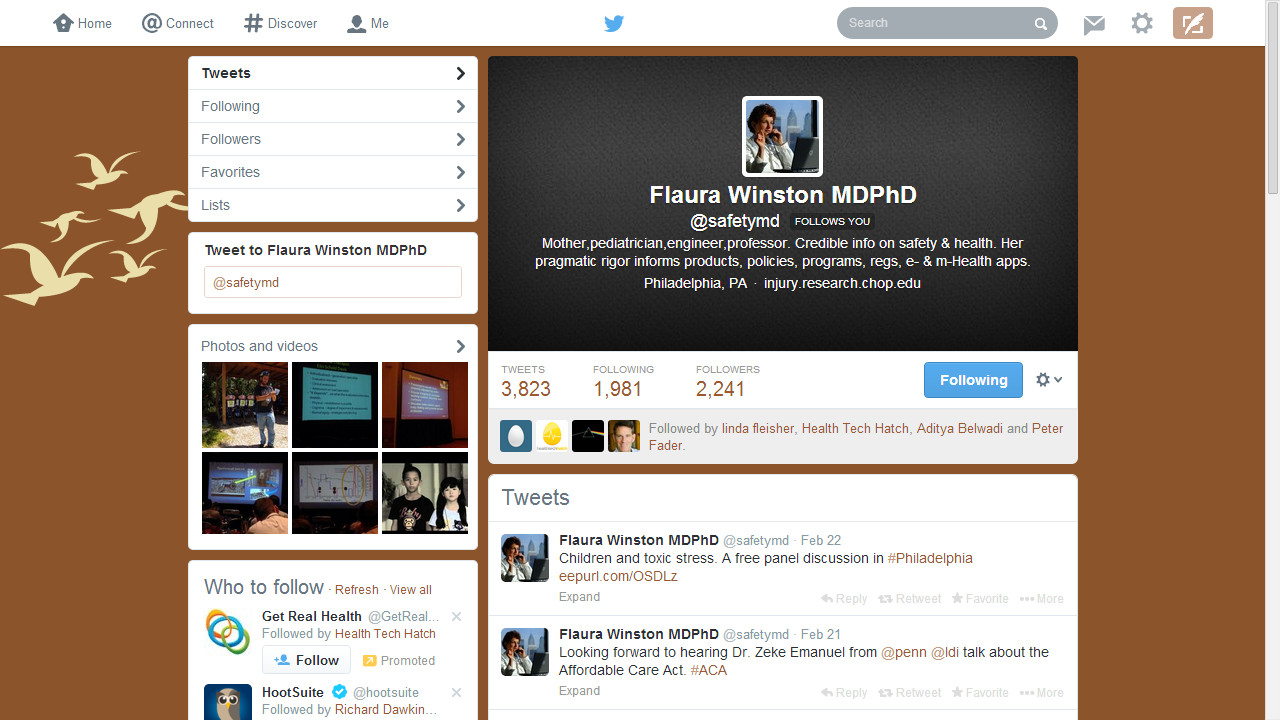
The @SafetyMD Twitter Page. Note: Screen shot was taken on February 16, 2014 (after this analysis had been conducted).

Content for @SafetyMD tweets and retweets is based on empirical research conducted within CIRP, new science from the injury research community at large, news, policy decisions, and advances in injury prevention strategies developed by industry. Followers of @SafetyMD include fellow physicians, nonprofit organizations, corporations, journalists, policymakers, and other influencers (eg, users from governmental or corporate entities) as well as researchers, entrepreneurs, and the general community. At the time of this analysis, @SafetyMD had 1790 followers (now 2550), and 458 tweets had been composed with an original-tweet-to-retweet ratio of 18 to 1 (indicating an emphasis on original content versus aggregated content). Given the purpose of the @SafetyMD handle, the relative diversity of its followers (in comparison to similarly sized research groups), and its level of activity, the authors concluded that @SafetyMD represented a typical academic/public health nongovernmental research entity that the CDC referred to in its Twitter strategy for disseminating health information. As such, @SafetyMD provided a convenient and generalizable platform to pilot test methods for measuring health information dissemination on Twitter. See Figure 1 for a screen shot of @SafetyMD’s current Twitter page.

### Data Extraction and Organization of the @SafetyMD Network

This protocol was exempt from review by the Children’s Hospital of Philadelphia’s Institutional Review Board. Based on Twitter protocols [6], we used the representational state transfer (REST) API version 1.0 provided by Twitter to acquire data. Specifically, we used the Status method to collect tweets posted by @SafetyMD and the Search method to collect retweets with keywords “RT *@username*.”

Between July and November 2012, available profile information (eg, handle name, total number of followers, number followed by, total number of original tweets, user-provided Twitter profile descriptions, and tweet/retweet text content) was obtained from the 1790 @SafetyMD followers. Using the REST API’s *GET statuses/user_timeline* method, a custom PHP-crawler program was developed to interface with the API, extract JavaScript Object Notation (JSON) data from followers, and store this data in a MySQL database for accessible tabular data formats. The resulting database included tweets from @SafetyMD’s network: those who follow @SafetyMD, those who retweet @SafetyMD, and those who retweet content from followers of @SafetyMD. Text content from @SafetyMD tweets was queried to identify the most frequent repeated words after the content was processed to remove stop words such as “a,” “and,” and “or.” [7]. In order to pilot test and evaluate the methodology, we extracted important keywords from the tweets to investigate how key information disseminated through the network. We employed term frequency, commonly used in information retrieval to measure a terms’ significance in the corpus, to identify as keywords terms repeated at least 10 times.

### Objective 1: Dissemination

This objective aimed to develop and apply methods for measuring the continued emphasis of health information themes as they spread through two levels of followers of @SafetyMD. A measure of user emphasis was defined as the proportion of total words in retweets that contained @SafetyMD keywords. As a baseline, we also measured the proportion of @SafetyMD keywords in the cumulative words of all @SafetyMD tweets. For this analysis, the @SafetyMD network was limited to those that were active retweeters and organized into three levels. In order to isolate mutually exclusive Twitter users, we defined Level 0 as @SafetyMD (n=458 original tweets), Level 1 as users who retweeted content from @SafetyMD (n=112 users, n=252 retweets), and Level 2 as those users who retweeted content from Level 1 and not directly from Level 0 (n=2356 users, n=4508 retweets).

### Objective 2: Targeted Interest Communities

In order to develop more targeted dissemination strategies, this objective examined whether @SafetyMD followers clustered into content-relevant groups. To pilot test this approach, a 10.00% random sample of @SafetyMD followers (n=179) was chosen and further limited to those who retweeted keywords from @SafetyMD (final sample analyzed n=170). For this sample, each user’s 50 most recent tweets (inclusive of original tweets, retweets from @SafetyMD, and retweets from others) were selected for content analysis, and available user profile information was extracted and linked to the content analysis. The range of each user’s 50 most recent tweets was set at 0 to 128 days from data extraction (this does not factor in each Twitter user’s account lifetime, which could fall within the 0-128 day range).

Unique words were identified from the sample of 8500 tweets by a custom-written Java program that removed handle names, URLs, punctuation symbols, and stop words. Words that included the hashtag symbol “#” as a prefix were kept because they represented a grouping of similar messages and topics on Twitter. This process yielded 1027 unique words. A word importance metric was created by computing the term frequency-inverse document frequency (TF-IDF) value using a Weka (Waikato Environment for Knowledge Analysis) filter class [8] for each word in each of the 8500 individual tweets. This processing generated a large matrix (170 followers × 1027 unique words) that required further data reduction through a 2-step process.

**Figure 2:**
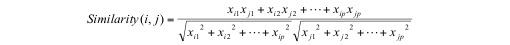
Formula calculating minimal distance.

First, principal component analysis (PCA) [9] was conducted to explain the variance-covariance structure of linear combinations of TF-IDF values. After excluding components with eigenvalues less than 1, a second step involved hierarchical cluster analysis (HCA) [10] on the components to further segment the sample of @SafetyMD followers. Similarity was calculated based on affinities among the components extracted from PCA. See Figure 2 for the formula calculating the minimal distance.

In this analysis, x_*ij*_= the TF-IDF value of term *j* in follower *i*. After calculating the distance between the new cluster and other clusters, HCA combined any two closest clusters recursively until the algorithm merged all the variables into one final cluster. The furthest neighbor (complete linkage) cluster method was used [10]. As an additional exploratory step, common interests from followers from each cluster were obtained by reading available Twitter profile descriptions and extracting common words and themes.

### Statistical Analysis

Descriptive statistics, including frequencies and proportions, were computed as appropriate. A scree plot and tabulated eigenvalues from the PCA and a dendrogram from the HCA were generated to identify unique clusters. All aggregate analyses were performed using R version 3.0.1 and SPSS version 19 (IBM Corp).

## Results

### Description of the @SafetyMD Network and Keywords

@SafetyMD had 1790 followers by the end of the 5-month study period and had composed 458 original tweets and retweets. The 1790 followers had a cumulative following base of 10,866,958 followers. The 458 @SafetyMD tweets and retweets contained 6538 words, and the stop word filter removed 2560 words (39.16%) of these words. A total of 31 keywords (words repeated at least 10 times) were generated from @SafetyMD’s 458 tweets, and these keywords were repeated 785 times (Table 1).

### Dissemination Across the Network

Of the 6538 words from the 458 tweets generated by @SafetyMD (Level 0), the keywords reflected a user emphasis, or proportion of @SafetyMD words in tweets that were keywords, of 12.01%. Within each subsequent level, the user emphasis remained and decreased: Level 1 contained 6.10% of @SafetyMD’s keywords among its 3711 retweeted words; Level 2, 2.50% of 60,795 retweeted words. All 31 keywords were represented in each level at least once.

The results depicted a possible dilution effect when retweeting from one level to the subsequent level. The @SafetyMD dissemination strategy aimed to use the viral nature of Twitter to spread evidence-based injury prevention information. While there was evidence of dissemination, only 2.50% of the content reached a second stage of spread. The change in keywords throughout the diffusion process demonstrated how users at different levels in the constructed retweet network could serve as a proxy filter. They pass on the core information posted by @SafetyMD while shifting the focus by disseminating other types of information such as social events and social behavior.

### Targeted Interest Communities

**Figure 3:**
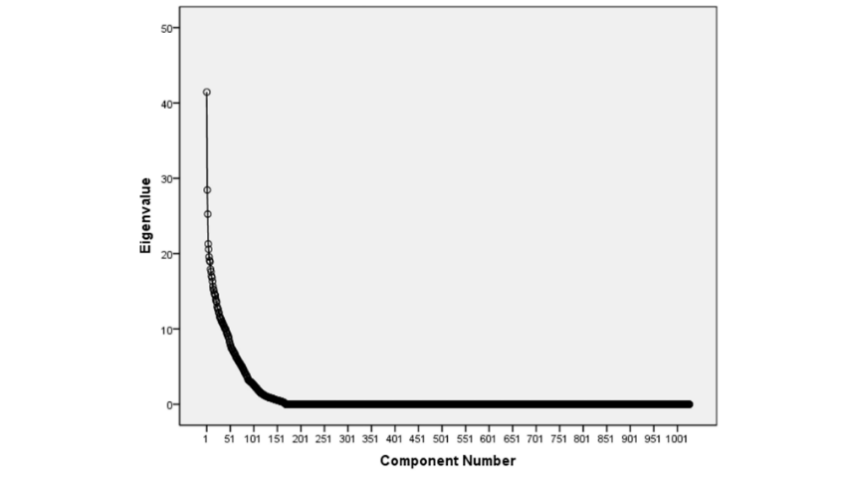
Scree plot of extracted components and their corresponding eigenvalues. Components with Eigenvalues <1 were excluded revealing 129 components.

The random sample was selected of 170 @SafetyMD followers who had composed 1,073,770 tweets and had a combined following base of 2,066,980 users. The 50 most recent tweets from these followers had a total word count of 35,602. Of these words, 9.60% were keywords, and all 31 @SafetyMD keywords were represented at least once among the 8500 tweets. PCA revealed 129 unique components from the data. Figure 3 depicts the scree plot generated from the 170×1027 matrix.

In an attempt to further classify the 170 followers, HCA generated 12 unique clusters from the 129 components (the dendrogram could not be presented due to its extremely large size but is available upon request). Table 2 reveals that the clusters shared common interests based on available Twitter profile descriptions provided by the users.

Nearly 60.0% of the followers were grouped in cluster 2 with common interests shared around driving education/safety and pediatric health and the words “drive,” “new,” “help,” “driver,” and “safe” had the five largest TF-IDF values. Although the HCA algorithm was able to differentiate clusters 5, 7, and 8, common interests from available Twitter profile descriptions were not available and could not be determined.

**Table 1:** @SafetyMD keywords.

Rank	Keyword	Frequency
1	#teendriving2012	112
2	teen	73
3	safety	51
4	driving	47
5	#safety2012	40
6	teens	32
7	driver	29
8	drivers	25
9	research	23
9	Safe	23
11	#roadsafety	21
12	#AMIA2012	20
12	#CelebrateMyDrive	20
12	CHOP	20
12	parents	20
16	crash	19
16	crashes	19
16	risk	19
19	chat	17
19	injury	17
21	car	16
22	study	15
23	drive	14
24	child	13
24	concussion	13
24	seat	13
27	#OHSU10X10	12
27	children	12
29	kids	10
29	passenger	10
29	positive	10

**Table 2:** Distribution of common interests across the 170 @SafetyMD followers grouped into 12 clusters.

Cluster	Followers, n	Common interests
1	5	Physician blogger, medical journalist
2	101	Driving school, drive training, driving safety, injury prevention, child health, pediatric physician
3	15	Moms, reporters, business owners
4	5	Spread of health-related information through social network
5	4	Could not be determined
6	8	Health research and health services, especially for driving safety
7	5	Could not be determined
8	6	Could not be determined
9	6	Physicians and medical research, especially child injury prevention
10	7	Medical education, driving education and training
11	4	Pediatricians and moms
12	4	Healthcare professionals, road safety professionals

## Discussion

### Principal Findings

As a popular microblogging platform, Twitter enables users to disseminate information to a large audience. Users can be selective in deciding whether to retweet a tweet, and this is a natural filtering process. Only those who consider the information as valuable and credible would pass along the message and make it accessible to peers with common interests. Although the information could be diluted throughout the information dissemination process, Twitter makes it easy and fast for information to reach more relevant people than the original author’s immediate network. This study is one of the first to develop methods for use by stakeholders to evaluate and optimize their use of Twitter for disseminating health information. Our newly developed methods provide preliminary evidence that individual stakeholders can evaluate the effectiveness of health information dissemination and create content-specific clusters for more specific targeted messaging through Twitter’s direct messaging function.

As an indicator of dissemination, the new metric *user emphasis* was defined representing the proportion of Twitter content that included keywords used by the stakeholder. The case study network, @SafetyMD, demonstrated a persistent but decreasing user emphasis of original @SafetyMD content (keywords) as the information spread through two levels of followers via retweeting. By Level 2 (the followers of @SafetyMD’s followers), user emphasis indicated that the use of @SafetyMD keywords had persisted but dropped by 9.5 percentage points. A second method created follower clusters based on content tweeted. The data reduction methods were able to differentiate 12 unique clusters of followers of @SafetyMD.

While multiple studies have conducted health information content analyses across Twitter networks [11-17], few have developed systematic methods to measure health information spread and leverage an existing Twitter network in order to differentiate interest groups. In a particular study utilizing NodeXL methods, Smith and colleagues [18] were able to identify clusters of content and proximal-based groups based on hashtags or selected words. In a study outside of the health sector examining the dissemination of anti-Islamic extremism on a popular Twitter handle, Blanquart and Cook [19] concluded that message dilution was a common phenomenon without the use of hashtags and embedded URLs in original tweets to magnify the messages. Our methods extend those reported previously by examining health information dissemination through a specific Twitter user network, identifying metrics for health information dissemination and leveraging an existing organization’s Twitter strategy to effectively reach targeted groups.

More than 10 years ago, Berwick [20] argued that health care leaders lag in translating successful scientific innovations into practice and provided 7 recommendations to accelerate the diffusion of innovations. More recently, Glasgow and colleagues [21] suggested that traditional implementation and dissemination strategies recommended by Berwick (such as getting packaged or messaged information to influencers via traditional networking and partnership) yield labor-intensive and cost-inefficient results. Kreuter and Bernhardt [22] extend these ideas and recommend that health care entities establish systematic evaluation measures to successfully disseminate evidence-based public health programs. The authors stress the need for more pragmatic methodologies for efficient dissemination of health care innovations. Our methods directly respond to this recommendation by providing tools for stakeholders to conduct systematic evaluation on their social media interventions.

Given the wide popularity of Twitter (nearly 232 million active users worldwide [23]), the CDC endorses its use as an opportunity to reach new audiences and bridge dissemination gaps [1]. However, health messages on Twitter can be lost in the large volume of content (more than 5000 tweets composed each second and nearly 300 billion cumulative tweets [23]). Our research supports the potential for Twitter to disseminate health information; however, Twitter communication strategies may need to be optimized.

### Limitations

Our results are not without limitations. The newly proposed indicator, user emphasis, only takes into consideration retweets across levels of followers within an existing network and does not account for original status updates that may reference @SafetyMD keywords. In addition, user emphasis may change over time based on new followers and the content that is shared. Also, the current calculation of user emphasis was limited to retweets resulting in a potential conservative estimate because it did not consider other ways that followers interacted with @SafetyMD (eg, through mentions and conversations). The list of the 31 @SafetyMD keywords was generated directly from @SafetyMD tweets and was not further compared to common words used in the broader health information environment. Therefore, we were not able to classify these keywords as original @SafetyMD content versus content influenced by the general health information environment on Twitter.

Our random selection of 170 @SafetyMD followers and their 50 most recent tweets may not necessarily represent content that best describes their information interests or needs, which might change over time. In particular, the range of recent tweet content was within 0 to 128 days of data extraction and may not have taken into account a user’s most current Twitter behavior. Future studies should examine a larger sample and the evaluation should be rolling over time to look at trends in interests. Also, the user-provided Twitter profile description might not have been current or complete (as it is part of the registration process and limited in length). Future studies might consider use of surveys to evaluate user interests.

It is clear from the HCA that the overwhelming majority of users were grouped in cluster 2; less than 10% were spread among the remaining 11 clusters. In addition, the common interests of many of the clusters are quite similar to each other, which may imply that available Twitter profile information is not a reliable and valid tool to describe mathematically unique groups on Twitter (given the inconsistency of available profile information). Future studies are needed to validate the relative uniqueness of these clusters and describe the clusters generated from this method using rigorous methodologies that may not involve using available profile information. Finally, these methods will only be useful as long as Twitter continues to share the data.

### Conclusions

This study aimed to develop and test a set of methods to (1) measure health information spread across an existing Twitter network and (2) leverage an existing Twitter network to identify target interest groups in an effort to provide tools for organizations that use Twitter to communicate health information. The results from @SafetyMD case study provide preliminary evidence of systematic yet simple tools that can be used to effectively leverage an existing Twitter network used to promote credible health information.

## Appendix

Multimedia Appendix 1 Peer-reviewed final report to funding agency (Pennsylvania Department of Health).

## Abbreviations

API: application program interface
CDC: Centers for Disease Control and Prevention
CIRP: Center for Injury Research and Prevention
HCA: hierarchical cluster analysis
HINTS: Health Information and National Trends Survey
PCA: principal component analysis
TF-IDF: term frequency-inverse document frequency
