# Supplementary material for: Measuring Health Information Dissemination and Identifying Target Interest Communities on Twitter: Methods Development and Case Study of the @SafetyMD Network
Source: JMIR Res Protoc. 2016 May 5;5(2):e50. doi: 10.2196/resprot.4203 (PMC4873622; doi:10.2196/resprot.4203)
Supplement: Multimedia Appendix 1 [file resprot_v5i2e50_app1.pdf]

FINAL REPORT

Project 2:
Methods to Evaluate the Content and Dissemination of Internet-Based Interventions to Prevent Injury

Project Period:
07/1/2009 – 12/31/2013

Principal Investigator:
Flaura K. Winston, MD, PhD

Institution:
The Children's Hospital of Philadelphia

This project was funded under a grant with the Pennsylvania Department of Health. The Department specifically disclaims responsibility for any analyses, interpretations or conclusions. Dr. Winston headed a team of investigators and staff in order to conduct this work without whom this work would not have been possible.

Investigators: Dr. Catherine McDonald, Dr. Chris Yang, Dr. Nancy Kassam-Adams, Dr. Yi-Ching Lee, Dr. Meghan Marsac, Dr. Linda Fleisher

Staff: Venk Kandadai, MPH; Kristen Kohser, MSW; Helen Loeb, PhD; Thomas Seacrist, MBE; Dan Bonfiglio. The PI and investigators would like to thank the Center for Injury Research and Prevention and its Simulator Program and Outreach Core; the Wharton Behavior Lab; and Pediatric Research Consortium at The Children's Hospital of Philadelphia. The authors also thank Donald Fisher, PhD, Daniel Mayhew, MA, Matthew Romoser, PhD. and Gerald Murphy for their assistance with this study. Most importantly, the PI and investigators thank the study participants who volunteered to advance knowledge about young driver crash risk.

We are proud to present a detailed report of our findings based on the milestones we originally proposed below.

Contents

CONTENTS...1

1. EXECUTIVE SUMMARY...1

1.1 Context.. 1
1.2 Specific Aims and Methods.. 1
1.3 Accomplishments, Findings and Conclusions... 2
2.1 Injury prevention: A major pediatric health issue
.... 4
2.2 Limits of established research methods and infrastructure 4
2.3 Research methods and infrastructure needed for injury prevention.. 4
2.4 Internet-based interventions to promote injury prevention.. 5
2.5 The Center for Injury Research and Prevention at The Children's Hospital of Philadelphia............. 5

3. SUMMARY OF THE RESEARCH PLAN................................6

Below find the specific aims and methods as outlined in the proposal. All aims were met and some were exceeded. Deliverables and results are presented in the later section... 6
3.1 Specific Aims.. 6
3.2 Research Design, Methods and Activities... 7

4. DETAILED REPORT ORGANIZED BY YEAR AND MILESTONES...10

4.1 Accomplishments for Years 1-2 (July 1, 2009 – June 30, 2011).. 11
4.2 Accomplishments Report for Year 3 Milestones:
July 1, 2011 – June 30, 2012.. 31

.... 31
4.3 Accomplishments Report for Year 4 Milestones:
July 1, 2012 – June 30, 2013.. 44
4.4 Accomplishments Report for Year 5 Milestones:
July 1, 2013 – December 31, 2013... 61

REFERENCES (DOES NOT INCLUDE REFERENCES FOR EMBEDDED REPORTS)..64

1. Executive Summary

1.1 Context

In Pennsylvania in 2007, 28% (374) of the 1,314 people killed in motor vehicle crashes were age 16- 24 years even though only 16% of the population over age 16 was in this age group. [1] To ensure the safety of our youth on the road, effective interventions are needed that engage youth where they interact – on the Web. However, in order for such interventions to prove effective, there is a crucial need to advance the development and application of rigorous methodologies for their development, evaluation and dissemination.

1.2 Specific Aims and Methods

Therefore, the overarching objective of this project was to establish rigorous methodologies for the systematic creation, evaluation and dissemination of Internet-based injury prevention interventions with an initial focus on interventions to reduce young-driver crashes and associated injuries. These methods were then applied to two primary research projects: (1) the evaluation of an evidence-based, Internet-delivered young driver safety intervention and (2) the evaluation of Twitter as a dissemination strategy for young driver safety evidence. As a result, the project was grouped into two research projects Specific aims were:

Aim 1: Create and Evaluate Internet-based interventions to prevent young-driver crashes
Method 1.1: Review best practices for creating and evaluating theoretically-grounded interventions to promote health and prevent injury, and adapt for Internet-based interventions to prevent young-driver crashes.
Method 1.2: Develop and implement a protocol to pretest content for Internet-based interventions and their component modules to assure that the interventions address the intended goals.
Method 1.3: Assess the feasibility of using a driving simulator for evaluating the efficacy of Internet-based interventions in changing driving performance.
Method 1.4: Test the relative efficacy of an evidence-based, Internet-delivered intervention (based on methods developed by the CHOP research team) as compared to that of a currently available intervention.

Aim 2: Establish a best practices methodology for dissemination of Internet-based interventions to prevent young-driver crashes, and methods to evaluate the impact of dissemination strategies
Method 2.1: Create best practice recommendations for dissemination of Internet-based interventions to prevent young-driver crashes.
Method 2.2: Create recommendations and metrics for evaluating dissemination of Internet-based interventions to prevent young-driver crashes (e.g., measuring reach, effectiveness, and unintended consequences).
Method 2.3: Create recommendations and metrics for evaluating dissemination of Internet-based interventions to prevent young-driver crashes (e.g., measuring reach, effectiveness, and unintended consequences).

1.3 Accomplishments, Findings and Conclusions

All milestones were met and/or exceeded. There were no changes in the aims.

This research project produced evidence-based frameworks, methods and metrics for the creation and dissemination of interventions to reduce young-driver crashes and their related injuries. Each of these takes the form of a Technical Report. In addition, a new, validated outcome measure to assess young driver safe driving skill was developed, the Simulated Driving Assessment. In particular, the following were produced:

1. An evidence-based framework for the development and evaluation of theoretically-grounded Internet-based interventions to promote safe behaviors among young drivers and their passengers.
2. An evidence-based protocol for intervention content pre-testing for young driver safety.
3. An evidence-based protocol to assess driving performance and behavior in a simulated environment (the Simulated Driving Assessment).
4. An evidence-based framework for the development of dissemination strategies for Internet-based interventions to promote safe behaviors among young drivers and their passengers.
5. An evidence-based framework for the evaluation of dissemination strategies via the Internet to promote safe behaviors among young drivers and their passengers.

These frameworks will serve as strong scientific foundations for the development and dissemination of interventions to prevent young-driver crashes and their related injuries.

In addition, below are the highlights of the products and findings from the research projects that were conducted as part of this grant.

- The Simulated Driving Assessment (SDA) created in this project is a new, safe, standardized measure of driving performance which taps key skill deficits and performance errors that lead to teen crashes. Results of this project demonstrated that the SDA is a valid stimulus to elicit differences in performance, and in driving skill, between novice teen and experienced adult drivers.
- A randomized trial of the Risk and Perception Training (RAPT) Internet-based intervention with newly licensed drivers revealed improvements in knowledge (as demonstrated by a statistically significant difference ($p<0.0001$) between baseline and post-intervention driving hazard anticipation scores for Trained teens (median=8; IQR=0). However, using the newly developed SDA, the proportion of teens having at least one crash during the entire SDA for the Untrained and Trained teens was 47% and 50% respectively ($p=0.87$). There was no statistically significant difference ($p=0.50$) in the distribution of total crashes for the Untrained (median=0; IQR: 0-1) and Trained (median=0.5; IQR: 0-2) teens.
- The methodology developed in this project included novel intervention evaluation methods and a novel outcome measure. Results demonstrated improvement in *knowledge* related to hazard anticipation, as evidenced by the improved scores post-RAPT training. However, the results from the SDA indicated that the teens did not

enact safety measures in high risk, complex driving scenarios. Thus, the SDA revealed a crucial gap between knowledge gained and translation to performance that prevents crashes. We discussed our findings with the lead investigator on RAPT and strategized ways to improve hazard anticipation training programs for novice teen drivers. The PA DOH grant allowed our team to develop a rigorous, standardized, valid assessment of driving performance that has implications beyond evaluation of hazard anticipation training.

- The results of our dissemination research study suggested that Twitter resulted in initial dissemination of messages (through @safetyMD) but very limited effectiveness in broad dissemination of safety messages and this might also be the case with other Twitter-based health information dissemination strategies. Hosting a Twitter chat is a strategy used by the Center for Injury Research and Prevention, the Centers for Disease Control and Prevention and others, which aims to enhance Twitter dissemination strategies. This project provided a rigorous evaluation of strategic use of a Twitter chat regarding teen driving. The Twitter chat resulted in initial traffic flow to an evidence-based website, TeenDriverSource.org, but did not result in sustained use or increasing visitors to the website. This technique resulted in only short-term improvements with no sustained changes. Rigorous methods such as those employed in this project are needed to hone social media strategies currently in widely recommended, so that researchers and policy makers can best target scarce time resources for effective dissemination of health and safety messages.

2. Overview and Context

Young driver crash prevention & Internet-based interventions to promote injury prevention

2.1 Injury prevention: A major pediatric health issue

Decades after two landmark National Academies' (NAS) studies emphasized its importance, injury remains the leading cause of death for persons ages 1-44 in the US. [2,3]. In 2004, there were 135,334 injury-related hospitalizations in Pennsylvania with charges exceeding $4.6 billion. A decade after an Institute of Medicine report [4]reiterated the NAS recommendations, the field of injury science has not developed (1) sufficient methodologies to develop and evaluate injury prevention and countermeasure strategies nor (2) infrastructures for implementation of proven strategies. In addition, injury prevention plays no obvious role in strategies to contain healthcare costs. Within the past decade, while other countries achieved substantial reductions in injury rates through concerted efforts, US injury mortality and serious injury rates increased. Children, youths, and young adults are the primary victims of injury, the leading cause of death and acquired disability in the young. *Pennsylvania can no longer sustain a health care system that relies on injury treatment as the primary strategy to reduce the burden of injury. Rather, our primary strategy must focus on achieving large scale dissemination of evidence-based prevention interventions.*

2.2 Limits of established research methods and infrastructure

Biomedical strategies to prevent disease rely on rigorous methods for drug/vaccine development that ensure safety and effectiveness. In contrast, such methods are not well-developed or widely applied to health promotion interventions. As a result, despite well-orchestrated and costly health promotion campaigns, confusion and poor compliance are common, with resultant limited effectiveness and unintended safety risks. *To ensure the safety and effectiveness of behavior change campaigns to prevent injuries, there is a crucial need to advance the development and application of rigorous methodologies for evaluating the use of information and its means of dissemination to achieve behavior change interventions, particularly those distributed widely via the Internet.*

2.3 Research methods and infrastructure needed for injury prevention

Young driver safety is a young field of inquiry, recently highlighted by the National Academies as an area that could advance greatly through the incorporation of state-of-the-art behavioral science. [5] However, enormous gaps exist in development and validation of methodologies to ensure effective creation and dissemination of injury prevention strategies for reducing young driver crashes. One key challenge involves the provision of a systematic outcome measure (beyond the infrequent and longer term outcome of driving citations and crashes). Driving simulators offer a safe alternative to on-road driving for the evaluation of performance. In addition, simulated drives allow for controlled manipulations of traffic situations producing a more consistent and objective assessment experience and outcome measure of crash risk. Yet, *few simulator protocols have been validated for their ability to assess driving performance under conditions that result in actual collisions.*

2.4 Internet-based interventions to promote injury prevention

The Internet holds excellent potential for advancing health and safety. Mass media has long been important for modifying attitudes, shaping behavior, and promoting health. However, despite vast reach, little effect of traditional media (newspapers, radio, and television) has been demonstrated on behavior change. [6-8] Rather, interpersonal channels have been more successful in influencing attitudes and motivating behavior change. [6,8] According to Cassell et al, “mass media channels are appropriate for creating awareness, but interpersonal interactions are essential for persuading individuals to adopt health-promoting behaviors.”

The Internet blends the *reach* of mass media with the effectiveness of *interpersonal interactivity*. According to the Pew Internet and American Life Project, the vast majority of Americans (88.5%) use the Internet, and the Internet serves as an important source of access to health information. [9] Disparities related to Internet access remain, but are steadily decreasing in recent years. According to Bennett et al, “Internet-based implementation allows participants to access intervention content at their convenience, in a manner that can feel largely anonymous. In contrast with other public health intervention approaches intended for large populations, Internet interventions can be structured to provide highly personalized messages, based on participant data. [10] The rich, interactive graphics available on the Web promote engagement.”

There can be considerable upfront development costs for comprehensive Internet interventions such as specialized websites, but their vast reach means that well-designed and disseminated web interventions can prove cost-effective. The efficacy of Internet interventions has been demonstrated across a wide range of health conditions. [11]

A major goal of this grant was to *create methods, particularly for Internet-based interventions*. In reviewing the literature, methods were needed across all phases of intervention research from *development to evaluation to implementation and broad dissemination*.
Once Internet-based interventions are proven effective, new methods can be used for implementation and broad dissemination. However, in writing this grant proposal, it was identified that few methods existed to evaluate implementation/dissemination strategies using new media (e.g., Twitter).

2.5 The Center for Injury Research and Prevention at The Children’s Hospital of Philadelphia

This research was conducted under the direction of Flaura Winston, MD PhD, the founder and Scientific Director of the Center for Injury Research and Prevention (CIRP). Dr. Winston oversees an established, highly successful and integrated 50-member team to realize the mission of CIRP: to advance the safety of children, youths, and young adults through research and action. The Center’s research-to-action-to-impact paradigm aims for wide dissemination of evidence by translating the Center’s scientific findings into actionable recommendations and information. Dissemination occurs through an established stakeholder network through the Web which includes members of the media, parents and communities, industry, government and other relevant practitioners.

The research activities of CIRP draw on a team of scientists from a broad range of fields including but not limited to medicine (emergency, pediatric, critical care, and adolescent), nursing (emergency, pediatric, critical care, and adolescent), health services research (epidemiology, biostatistics, and demography), surgery (general, pediatric, orthopedic), psychology (developmental, pediatric, clinical, social, neuropsychology, and trauma), engineering (bio- and automotive engineering and field investigation), public health (knowledge transfer, education, communication, community- based participatory research), and social sciences (anthropology, qualitative studies, focus group conduct, interviewing, survey design and analysis, sociology). The Center's scientific capabilities are complemented by an outreach team that includes experts in health education, technical writing, media relations, community mobilization, health marketing, and video and Web production to achieve its mission. This project leveraged and extended the capabilities of the Center to meet the aims.

3. Summary of the Research Plan

Below find the specific aims and methods as outlined in the proposal. All aims were met and some were exceeded. Deliverables and results are presented in the later section.

3.1 Specific Aims

Aim 1: Create and Evaluate Internet-based interventions to prevent young-driver crashes

Method 1.1: Review best practices for creating and evaluating theoretically-grounded interventions to promote health and prevent injury, and adapt for Internet-based interventions to prevent young-driver crashes.

Method 1.2: Develop and implement a protocol to pretest content for Internet-based interventions and their component modules to assure that the interventions address the intended goals.

Method 1.3: Assess the feasibility of using a driving simulator for evaluating the efficacy of Internet-based interventions in changing driving performance.

Method 1.4: Test the relative efficacy of an intervention (based on methods developed by the CHOP research team) as compared to that of a currently available intervention.

Aim 2: Establish an evidence-based methodology for marketing or dissemination of Internet-based interventions to prevent young-driver crashes and for real world evaluation of their use

Method 2.1: Create best practice recommendations for dissemination of Internet-based interventions to prevent young-driver crashes.

Method 2.2: Create recommendations and metrics for evaluating dissemination of Internet-based interventions to prevent young-driver crashes (e.g., measuring reach, effectiveness, and unintended consequences).

Method 2.3: Create recommendations and metrics for evaluating dissemination of Internet-based interventions to prevent young-driver crashes (e.g., measuring reach, effectiveness, and unintended consequences).

3.2 Research Design, Methods and Activities

3.2.1 (Aim 1): Create and Evaluate Internet-based interventions to prevent young-driver crashes

3.2.1.1 (Method 1.1): Review literature for creating and evaluating theoretically-grounded interventions to promote health and prevent injury, and adapt for Internet-based interventions to prevent young-driver crashes

Rationale: Successful behavior change approaches rely on rigorous methods and an underlying theoretically-grounded model to 1) define an evidence-based strategy, 2) choose among the many possible intervention components, and 3) evaluate both the process and outcomes of interventions as they are implemented. [12] A theoretically-grounded, evidence-based behavior change model is built by first defining a broad measurable vision (e.g., adoption of a behavior that will reduce injury risk) and then progressively narrowing in focus to identify smaller component goals (e.g., changes in attitudes, skills, attitudes or knowledge that will increase adoption of the behavior). [13,14] Prevention strategies are then created to address these component goals; strategies can be aimed at the population, an individual, or both. Internet-based behavior change interventions are relatively new and the methods and models to support this work are not well-developed. [11]
For this method, we followed these steps:
Step 1 – Consulted with experts and conducted a literature review to identify and integrate current published and non-published knowledge about the development of Internet-based interventions for young driver safety.
Step 2 - Created an evidence-based, systematic framework for the development of theoretically-grounded Internet-based interventions to promote safe behaviors among young drivers and their passengers. The framework was created such that interventions developed based on this framework will be better able to be evaluated in rigorous trials.

3.2.1.2 (Method 1.2): Develop and implement a protocol to pretest content for Internet-based interventions and their component modules to assure that the interventions address the intended goals.

Rationale: Paths of influence to achieve behavior change begin with smaller addressable component goals (e.g., changes in attitudes, skills, behaviors, knowledge, or perceived norms that increase the likelihood that a safe behavior will be adopted). Rigorous development of intervention content must include pre-testing with the target audience to

assure that the intervention addresses the intended goals, followed by informed, iterative modification and evaluation of content before implementation.

For this method, we followed these steps:
Step 1 – Consulted with experts and conducted a literature review to identify and integrate current published and non-published knowledge in pre-testing of Internet-based interventions for young driver safety.
Step 2 - Created an evidence-based, systematic protocol for intervention content pre-testing of Internet-based interventions for young driver safety.

3.2.1.3 (Method 1.3): Assess the feasibility of using a driving simulator for evaluating the efficacy of Internet-based interventions in changing driving performance.

Rationale: The key outcomes of interest for interventions aimed at young drivers are driving behavior and performance. One major challenge for rigorous evaluation of teen driver interventions is that the inherent hazard of on-road driving makes it difficult to safely evaluate changes in driving behavior. Advanced driving simulators provide a safe means for collecting objective data on driver proficiency, and allow better control of traffic scenarios whose influence on behavior is the target of evaluation. [15-17] Simulators also allow rapid evaluations of intervention outcomes (driving behaviors in specific scenarios) without observing lengthy periods of on-road experience. Simulated environments can be adapted to nighttime driving, or to driving with peer passengers, in order to observe how young drivers behave under these and other known hazardous conditions. Validation of these assessment methods for young drivers is necessary to ensure that the simulated environments reflect real-world performance.

We developed and validated a simulator-based protocol for assessing young driver performance in evaluation studies (Simulated Driving Assessment), via the following steps:
Step 1 – Selected and purchased a high fidelity, commercially available driving simulator and set up simulator for use with young drivers.
Step 2 – Consulted with experts and practitioners and reviewed the literature on driver training and evaluation to identify available tools for assessment of driving performance.
Step 3 – Developed an evidence-based protocol (the SDA) to assess driving performance and behavior in a simulated environment.

In addition, a validation study of the Simulated Driving Assessment was conducted as no evidence-based protocol was discovered.

3.2.1.4 (Method 1.4): Test the relative efficacy of an intervention (based on methods developed by the CHOP research team) as compared to that of a currently available intervention with respect to changes in driving behavioral variables.

In keeping with the methods proposed in our application, the intervention to be investigated was determined after the methods above were developed. We reviewed the literature to find an intervention whose development was consistent with the guidelines established in

Method 1.1. Pre-testing of the intervention was conducted with a small number of pilot participants. An intervention trial was conducted to test the following hypothesis: When compared to a standard intervention (in this case – usual case young driver preparation through the learner phase), the chosen intervention will result in improved knowledge (as evidenced by a post-test) and improved safe driving performance (as evidenced by performance on the Simulated Driving Assessment).

3.2.2 (Aim 2): Establish an evidence-based methodology for marketing or dissemination of Internet-based interventions to prevent young-driver crashes and for real world evaluation of their use

3.2.2.1 (Method 2.1): Create best practice recommendations for dissemination of Internet-based interventions to prevent young-driver crashes.

Rationale: Given interventions with proven efficacy and effectiveness, achieving public health impact requires a systematic, evidence-based marketing and dissemination strategy to reach broad audiences and promote behavior adoption. Established non-Internet-based research methods exist to identify dissemination channels and create marketing strategies for interventions. [18] Marketing and dissemination encompass a wide range of potential activities including but not limited to direct to user advertising, dissemination and awareness among influencers of the users, improved delivery to promote and improve ease in use and other methods for optimizing awareness, demand, delivery and use. The Internet provides new partners and new dissemination channels for evidence-based intervention delivery (e.g., bloggers as partners and social media networks as dissemination channels). Sustainability of health marketing strategies requires research to determine on-going costs and to identify the partners (among businesses, private non-profits and government agencies) who will most likely benefit from on-going investment.

For this method, we followed these steps:
Step 1 – Consulted with experts and conducted a literature review to identify and integrate current published and non-published knowledge in Internet-based health promotion and prevention marketing or dissemination applicable to young driver safety promotion. In conducting this step, we identified Dr. Chris Yang at Drexel University and collaborated with him for the remainder of this aim.
Step 2 - Created an evidence-based framework for the development of marketing or dissemination plans for Internet-based interventions to promote safe behaviors among young drivers and their passengers. The framework was created such that interventions developed based on this framework can be evaluated in rigorous trials.

3.2.2.2 (Method 2.2): Create recommendations and metrics for evaluating dissemination of Internet-based interventions to prevent young-driver crashes (e.g., measuring reach, effectiveness, and unintended consequences).

Rationale: Empirical studies evaluating large scale marketing or dissemination of public health programs are limited both in number and quality. [19] Ideally, evaluations are conducted to assess whether revisions to the marketing or dissemination plans are needed (e.g., see section 1). Public health campaigns delivered via the Internet provide opportunities for sustainable, affordable on-going tracking and evaluation not available to traditional, non-Internet-based campaigns. These tracking and evaluation methods are new (e.g., www.google.com/analytics) or under development and there is limited experience in their use for health and safety promotion campaigns. Examples of evaluation measures might include: reach (e.g., metrics for the diffusion of an intervention via links and referrals or time spent viewing on-line components); sustainability (e.g., metrics over time); and costs (of monitoring and revisions).

For this method, we followed these steps:
Step 1 – Consulted with experts and conducted a literature review to identify and integrate current published and non-published knowledge in methods and metrics around measurement of actual use patterns for Internet-based health and safety interventions as applied to young driver safety promotion.
Step 2 - Created an evaluation framework to assess the actual use patterns for Internet-based interventions for the promotion of young driver safety. The framework provides metrics and methods that can used in rigorous evaluation trials of Internet-based interventions and their promotion.

3.2.2.3 (Method 2.3): Create recommendations and metrics for evaluating dissemination of Internet-based interventions to prevent young-driver crashes (e.g., measuring reach, effectiveness, and unintended consequences).

We chose our Center's evidence-based website, TeenDriverSource.org as an intervention whose development was consistent with the guidelines established in Aim 1. A marketing campaign based on Twitter (i.e., a "Twitter chat") was chosen as such a strategy was consistent with Methods 2.1 and 2.2. An intervention trial of the effectiveness of the Twitter chat was conducted. The intervention trial tested the following hypothesis: When compared to a standard marketing or dissemination plan (i.e, no targeted outreach for the period immediately before the Twitter chat), the Twitter chat would result in increased visits to TeenDriverSource.

4. Detailed Report Organized by Year and Milestones

Below, we report on milestones as they pertain to the project in chronological order (Project Timeline: July 1, 2009 – December 31, 2013) All milestones were completed and no aims were changed.

4.1 Accomplishments for Years 1-2 (July 1, 2009 – June 30, 2011)

4.1.1 Milestones for Year 1 (July 1, 2009 – June 30, 2010)
(Note: Project started on January 1, 2010)

**Milestones**	**Project Aims Satisfied**	**Annual Report Submission**
1. Start expert interviews and literature reviews	1, 2	7/1/2009-6/30/2010
2. Select and purchase a high fidelity, commercially available driving simulator and set up simulator for use with young drivers	1	7/1/2009-6/30/2010

4.1.2 Milestones for Year 2 (July 1, 2010 – June 30, 2011)

**Milestones**	**Project Aims Satisfied**	**Annual Report Submission**
1. Complete expert interviews and literature reviews and identify and adapt methods as described above	1, 2	7/1/2010-6/30/2011
2. Develop protocol for evaluating young driver behavior in the simulator	1	7/1/2010-6/30/2011
3. Design study to evaluate methods to develop young driver safety interventions	1	7/1/2010-6/30/2011

The overriding goal of the Tobacco-settlement-funded project was to create systematic methods for the development and evaluation of Internet-based interventions. These milestones involved extensive literature reviews that were summarized into reports and template protocols, filling a gap for the field.

4.1.3 Accomplishments for Year 1, Milestone 1: Start expert interviews and literature reviews

All procedures were initiated on-time as outlined in the proposal methods

4.1.4 Year 1, Milestone 1 & Year 2, Milestone 3: Final Report on methods for developing and evaluating internet-based interventions for young drivers

For these milestones, two technical reports were submitted in Year 2 that capture the accomplishments. The first technical report is included in its entirety below. The second report is summarized and the template protocol is included in the Appendix.

4.1.5 Technical Report: Framework for developing theoretically-grounded Internet-based interventions (with emphasis on promotion of interventions for safe behaviors among young drivers and their passengers, includes review of literature and expert interviews)

Overview

While much attention has been given to the evaluation of Internet-based interventions (Calear, Christensen, Mackinnon, Griffiths, & O'Kearney, 2009; Marks, Cavanagh, & Gega, 2007; L. Ritterband & Tate, 2009; Lee M. Ritterband, et al., 2003) as well as the dissemination of Internet based programs(Cho, 2003; Crutzen, 2009; Crutzen, et al., 2008, 2009; Duffy, 2000; Morahan-Martin, 2004; Previte, 2005; Ramo, Hall, & Prochaska, 2010; Roche & Skinner, 2009; Rothbaum, Martland, & Jannsen, 2008; Scullard, Peacock, & Davies, 2010; Van Deursen & Van Dijk, (in press); Weaver, et al., 2010) , best practices for the development of Internet-based interventions have yet to be established. Kassam-Adams and colleagues (2010) and Ritterband et al. (2009) provide suggestions for developing health-based (physical and psychological) websites which can be adapted more generally for the creation of Internet-based interventions aimed to influence behaviors. Kassam-Adams and colleagues note that there are several critical decision points when initiating the development of an Internet-based intervention including the defining the purpose of the intervention, the intended population, and the design or look and feel of the intervention(Kassam-Adams, Marsac, & Winston, 2010). Ritterband et al (2009) suggest considering nine dimensions in developing Internet-based behavioral change programs including user characteristics, environmental factors, website use and program adherence, support and website characteristics, behavior change, symptom improvement and treatment maintenance(Lee M. Ritterband, Thorndike, Cox, Kovatchev, & Gonder-Frederick, 2009). Informed by Kassam-Adams et al (2010) and Ritterband et al.'s (2009) suggestions, we provide a series of steps to serve as a guideline for the development of Internet-based interventions (See Table 1 for a summary of steps). This guide can be applied to the development of Internet-based interventions across topics and is particularly relevant to Internet-based programs designed to create behavioral change. Throughout this guide, we provide an example of applying this guide to the development of an Internet-based intervention for promoting safe behaviors among young drivers and their passengers.

Step 1: Identify and explicitly state problem and purpose of the intervention

Clearly describe the problem present and the intention of the intervention to ensure the intervention has a clear path and to select relevant mechanisms for change (L. M. Ritterband & Thorndike, 2006).

Example: Teen drivers (ages 16 to 19) are involved in fatal crashes at four times the rate of adult drivers (ages 25 to 69), particularly during the first six months after licensure, indicating that a substantial amount of learning occurs during this time. The inexperience problem is largely related to deficient practice with adult supervision. Research has shown that driver's education classes and parent supervised practice are not providing teens with the quantity, quality, and variety of training needed to develop skills to be safe behind the

wheel. The purpose of the intervention is to decrease motor vehicle crashes and related injuries by increasing safe behaviors in teenage drivers and their passengers.

Step 2: Linking intervention goals to theory of change

In defining the purpose of the intervention, it can be helpful to select a theory to guide the direction of the intervention (Benight, Ruzek, & Waldrep, 2008). Program theory (e.g., Rogers, Petrosino, Huebner, & Hacsi, 2000) is one example of a useful framework for developing content for interventions (Winston & Jacobsohn, 2010), which is easily applied to Internet interventions (Kassam-Adams, et al., 2010; Winston & Jacobsohn, 2010). Working backwards, one can first identify key outcomes, then suggest target constructs that will lead to those key outcomes, and finally design specific content tailored to the selected target constructs (Kassam-Adams, et al., 2010; Winston & Jacobsohn, 2010) See Winston and Jacobsohn (2009) for a detailed explanation of applying program theory to behavioral interventions(Winston & Jacobsohn, 2010).

Example: Figure 1 provides an example of program theory as applied to promoting safe driver and passenger behavior via an Internet intervention.

Figure 1.* The application of program theory to Internet-interventions to increase safe driving behavior in novice drivers

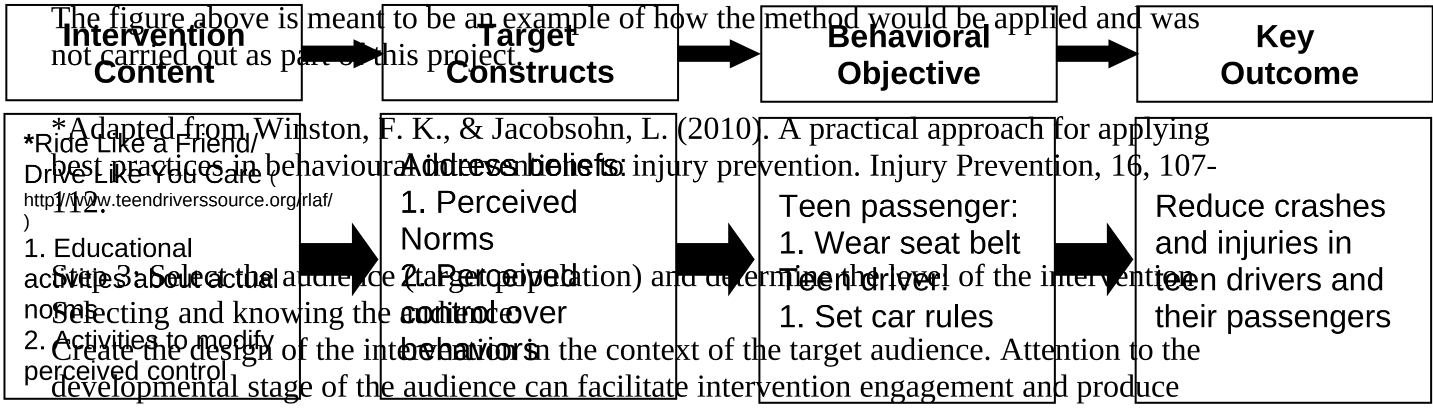

The figure above is meant to be an example of how the method would be applied and was not carried out as part of this project.

*Adapted from Winston, F. K., & Jacobsohn, L. (2010). A practical approach for applying best practices in behavioural interventions to injury prevention. Injury Prevention, 16, 107-112.

Step 3: Select the audience (target population) and determine the level of the intervention

Selecting and knowing the audience:

Create the design of the intervention in the context of the target audience. Attention to the developmental stage of the audience can facilitate intervention engagement and produce better intervention outcomes. Including members of the audience in the initial intervention plans can help in this process (Winston & Jacobsohn, 2010).

Example: The intended population for promoting safe driving behavior in youth can be the new drivers, youth passengers, parents, driving instructors, schools or other community members. In the example above, the audience would be young drivers. Thus, consultation with individuals who specialize in adolescent development may be helpful in knowing how to engage adolescents in the intervention. Additionally, including several new, adolescent drivers on the team can help ensure that the intervention is appealing to adolescents.

Identifying the level of the intervention:
The level of the intervention is determined by the goals and intervention components. Selecting the level of the intervention helps to guide the types of activities/intervention components that are developed. Adapting from Kassam-Adams and colleagues (2010)

suggestions of levels of websites for trauma-related web programs, Internet interventions can be categorized into three levels:

Level 1: Informational (providing facts, explanations, links to resources)
Level 2: Psychoeducational intervention/universal (providing education and skills practice to change behaviors or behavioral intentions across all individuals in the population)
Level 3: Problem-focused/Targeted (identifying a specific problematic behavior and intervening with specifically identified individuals).

Example: In the example detailed out in program theory (above), the intervention is psychoeducational. The intervention extends beyond the provision of information to include activities to change adolescents' thinking and behaviors.

Step 4: Identify potential barriers of intervention implementation
In collaboration with end users, identify barriers of intervention implementation and problem-solve solutions (Winston & Jacobsohn, 2010). Interviews or open-ended questions are an easy way to obtain this information.

Example: In implementing an Internet intervention for early drivers and passengers possible barriers include access to the Internet, parental consent, and willingness of teenagers to participate. Solutions could include requesting driver's education classes or schools to provide time and access to computers to implement the program. Parental consent could possibly be obtained through the school or when adolescents' apply for their learner's permit.

Step 5: Consider evaluation
Ask the question, how will we know if the intervention is achieving its intended behavioral objectives and overall goal? If it is unclear about how to evaluation the intervention, re-visit the program theory to determine if adjustments are needed to create measurable goals (Winston & Jacobsohn, 2010).

Example: A research study could examine adolescents' beliefs before and after participating in the program using survey measures. Beliefs about driver and passenger safety behaviors could be compared between adolescents completing the "Ride Like a Friend, Drive Like You Care" program to those who did not complete the program. Driver and accident records could be examined and compared between groups to evaluate the overall program goal.

Step 6: Draft content to match theory
After the theory and content of the intervention have been established, consider integrating features which have proven successful Internet-based intervention such as sound, graphics, and interactive activities (Lee M. Ritterband, et al., 2006).

Example: "In Ride Like a Friend, Drive Like You Care," the website was designed using input from teenagers and integrating videos (e.g., "Teen on the Street Questions") and presenting statistics with pictures and graphs.

Step 7: Build intervention prototype and complete usability/functionality testing & revise intervention to maximize functionality
Usability is essential since the intervention cannot attain its goals if people do not understand how to use it (Kassam-Adams, et al., 2010).

Example: To evaluate “Ride Like a Friend, Drive Like You Care,” researchers could ask a group of teens to find answers to specific questions or to watch videos to obtain specific information. If participants have a difficult time finding the information, then the intervention would need to be revised for functionality.
Step 8: Evaluate intervention implementation and revise if indicated
Conduct a full-scale evaluation. Continue to evaluate intervention engagement, user satisfaction, as well as behavioral objectives and intended outcomes.

Example: To evaluate “In Ride Like a Friend, Drive Like You Care,” both the behavioral objectives and target outcomes should be evaluated (see Step 5 for examples of how this intervention would be evaluated).

Table 1. Recommended steps for developing Internet-based interventions

Steps	Questions to ask
Step 1: Identify and explicitly state problem and purpose of the intervention	What is the problem that needs addressed? What specific behaviors can we target to address the problem?
Step 2: Linking intervention goals to theory of change	What skills/knowledge are needed to achieve the goals of the intervention? What are the current techniques/theories in the field that can be implemented?
Step 3: Select the audience (target population) and determine the level of the intervention	Who do we want to complete the intervention? Is this intervention beyond information provision? Is this intervention for anyone or are we targeting people who are already at-risk for difficulties?
Step 4: Identify potential barriers of intervention implementation	What would get in the way of someone being able to use this intervention? What are barriers for someone completing this intervention?
Step 5: Consider evaluation	How will we measure our intervention behavioral objectives and goals? Are there proximal and distal outcomes to consider?
Step 6: Draft content to match theory	What activity can teach the knowledge or skills needed to solve our identified problem and meet our intervention goals? Are there effective techniques currently that can be translated to an Internet-based delivery system? What features of Internet interventions have worked for others?
Step 7: Build intervention prototype and complete usability/functionality testing & revise intervention to maximize functionality	Would users be interested in this type of an intervention? What would encourage them to use this? What would prevent them from engaging in this program? Are there any cultural factors to consider? What should the design look like? Are members of the target population able to find the information or complete tasks without additional assistance?
Step 8: Evaluate intervention implementation and revise if indicated	What the intervention implemented as intended (guided by theory)? If not, why not? Were the behavioral objectives and goals attained?

REFERENCES

Benight, C. C., Ruzek, J. I., & Waldrep, E. (2008). Internet interventions for traumatic stress: A review and theoretically based example. [Article]. Journal of Traumatic Stress, 21(6), 513-520.
Calear, A. L., Christensen, H., Mackinnon, A., Griffiths, K. M., & O'Kearney, R. (2009). The YouthMood Project: A cluster randomized controlled trial of an online cognitive behavioral program with adolescents. Journal of Consulting and Clinical Psychology, 77(6), 1021-1032.
Cho, C. H. (2003). Factors influencing clicking of banner ads on the WWW. Cyberpsychol Behav, 6(2), 201-215.
Crutzen, R. (2009). Hard to Get, Hard to Keep: Dissemination of and exposure to Internet-Delivered Health Behaviour Change Interventions Aimed At Adolescents. Unpublished Dissertation, Maastricht University, Maastricht.
Crutzen, R., de Nooijer, J., Brouwer, W., Oenema, A., Brug, J., & de Vries, N. K. (2008). Internet-delivered interventions aimed at adolescents: a Delphi study on dissemination and exposure. Health Educ. Res., 23(3), 427-439.
Crutzen, R., de Nooijer, J., Brouwer, W., Oenema, A., Brug, J., & de Vries, N. K. (2009). A conceptual framework for understanding and improving adolescents' exposure to Internet-delivered interventions. Health Promot. Int., 24(3), 277-284.
Duffy, M. (2000). The Internet as a research and dissemination resource. Health Promot. Int., 15(4), 349-353.
Kassam-Adams, N., Marsac, M. L., & Winston, F. K. (2010). Preventing traumatic stress after child injury: Development of a website for parents. In A. Brunet & R. Andrea (Eds.), Internet Use in the Aftermath of Trauma (pp. 157-178): IOS Press.
Marks, I. M., Cavanagh, K., & Gega, L. (2007). Computer-aided psychotherapy: revolution or bubble? The British Journal of Psychiatry, 191(6), 471-473.
Morahan-Martin, J. M. (2004). How Internet Users Find, Evaluate, and Use Online Health Information: A Cross-Cultural Review. [Article]. CyberPsychology & Behavior, 7(5), 497-510.
Previte, J. (2005). Understanding everyday internet experiences: Applications to social marketing theory and practice. Queensland University of Technology.
Ramo, D. E., Hall, S. M., & Prochaska, J. J. (2010). Reaching young adult smokers through the Internet: Comparison of three recruitment mechanisms. Nicotine & Tobacco Research, 12(7), 768-775.
Ritterband, L., & Tate, D. (2009). The Science of Internet Interventions. Annals of Behavioral Medicine, 38(1), 1-3.
Ritterband, L. M., Cox, D. J., Gordon, T. L., Borowitz, S. M., Kovatchev, B. P., Walker, L. S., et al. (2006). Examining the Added Value of Audio, Graphics, and Interactivity in an Internet Intervention for Pediatric Encopresis. [Article]. Children's Health Care, 35(1), 47-59.
Ritterband, L. M., Gonder-Frederick, L. A., Cox, D. J., Clifton, A. D., West, R. W., & Borowitz, S. M. (2003). Internet interventions: In review, in use, and into the future. Professional Psychology: Research and Practice, 34(5), 527-534.
Ritterband, L. M., & Thorndike, F. (2006). Internet Interventions or Patient Education Web Sites? . Journal of Medical Internet Research, 8(3), e18.

Ritterband, L. M., Thorndike, F. P., Cox, D. J., Kovatchev, B. P., & Gonder-Frederick, L. A. (2009). A behavior change model for Internet interventions. Annals of Behavioral Medicine, 38(1), 18-27.

Roche, M., & Skinner, D. (2009). How Parents Search, Interpret, and Evaluate Genetic Information Obtained from the Internet. Journal of Genetic Counseling, 18(2), 119-129.

Rothbaum, F., Martland, N., & Jannsen, J. B. (2008). Parents' reliance on the Web to find information about children and families: Socio-economic differences in use, skills and satisfaction. [doi: DOI: 10.1016/j.appdev.2007.12.002]. Journal of Applied Developmental Psychology, 29(2), 118-128.

Scullard, P., Peacock, C., & Davies, P. (2010). Googling children's health: reliability of medical advice on the internet. Archives of Disease in Childhood, 95(8), 580-582.

Van Deursen, A., & Van Dijk, J. ((in press)). Using the Internet: Skill related problems in users' online behavior. Interacting with Computers.

Weaver, J. B., III, Mays, D., Weaver, S. S., Hopkins, G. L., Eroglu, D., & Bernhardt, J. M. (2010). Health Information-Seeking Behaviors, Health Indicators, and Health Risks. Am J Public Health, 100(8), 1520-1525.

Winston, F. K., & Jacobsohn, L. (2010). A practical approach for applying best practices in behavioural interventions to injury prevention. Injury Prevention, 16, 107-112.

4.1.6 Technical Report: Protocol for intervention content pre-testing of Internet-based interventions (with emphasis on promotion of interventions for safe behaviors among young drivers and their passengers, includes review of literature and expert interviews) (includes review of literature and expert interviews)

Technical Report 1, included above, presented the summary of our extensive literature and expert review regarding evidence-based, rigorous development and evaluation of Internet-based interventions for the safety of young drivers and their passengers. One key step in intervention development involves the testing of content with the target audience, in this case the target audience would be adolescents and the venue for the delivery of the content is the Internet. No such protocol to conduct this work existed and, therefore, to advance our future work and the field, a template protocol was created to conduct this work. As proposed, this protocol advances rigorous development of effective interventions and aids intervention developers by creating systematic methods to test content. It is a necessary initial step in studies in which interventions are being developed de novo.

The Template Protocol for message / content testing when designing new interventions is included in the Appendix. This template protocol serves as a ready-to-use IRB protocol that will aid future investigators in employing systematic message-testing. For this project, these methods were incorporated into the pilot phase of the RAPT evaluation study.

4.1.7 Report on the selection and purchase of a high fidelity, commercially-available driving simulator (in fulfillment of Year 1, Milestone 2)

A high fidelity, commercially available, multiple-computer driving simulator system was purchased. The driving simulator makes use of an actual partial vehicle cab that has been modified to include instrumentation and audio cueing systems (see Figure 1). Three forward 46" liquid crystal display (LCD) panels display simulated driving environment extending approximately 120° field of view. The cab and the simulated scene together create an immersive visual environment at a resolution of 1280x1024. Using the actual cab structure assures the maximum amount of realism and accuracy with respect to the locations of controls, driver visibility, and feel of the desired vehicle. All electrical components in the center console remain functional. Audio cueing components such as speakers and vibration transducers are integrated into the cab to further support the simulation. This cab includes the following controls and results in the appropriate vehicle behavior in the simulator:

Control loaded steering wheel (forced feedback steering system)
Throttle (active feedback system)
Brake (active feedback system)
Gear selector for automatic transmission (P, R, N, D, L1, L2)
Turn signals

Seat belt
Headlights switch
Ignition switch (status only)

The dash indicators will respond to the status of the simulated vehicle and may be controlled from the scenario control system. These indicators consist of the following gauges:

Speedometer
Turn signal indicator
Engine oil pressure
Fuel level
Warning lights

Visual rendering and scenario control is supported by real-time driving simulation software. This software manages the communication between all of the relevant subsystems including visuals, scenario control, audio, motion, control loading, and data collection. Data is collected at 60 Hertz (Hz).

The simulation environment is programmed by placing tiles of roads, roadway objects, and scenario control objects. Tiles are generally 200 meters by 200 meters in size. Once the tiles required to support a given scenario are in place, additional features such as trees, signage, buildings, etc. can be added. Other features such as traffic signal controls, pedestrians, vehicles, and other dynamic elements can be added to create representative driving experience. A set of special objects called scenario control objects are then added to control the actions and behaviors of the vehicles, pedestrians, and other controllable features within the simulator. Scenario control objects are sensors, markers, paths, start points, and end points, etc. Some of these objects have a script attached to them that allows the experimenters to issue commands to the simulator in order to make certain behaviors happen during the simulation, such as making a pedestrian walk or making a lead vehicle brake at a pre-determined location.

Once scenario design is completed, research participants can sit in the driver's seat and drive the scenario by looking at the simulated environment and controlling the vehicle while interacting with the dynamic features in the environment. For example, one scenario could have pedestrians run across the road unexpectedly and test how the research participant reacts to such hazards.

Five cameras are mounted in the driving simulator system to record drivers' performance. This video capture and review system allows capturing and playing back synchronized views of a simulation experience along with recorded data. The system records simulation activity from up to four vantage points. These views are simultaneously displayed in quad view upon play back. In addition, the video review system supports preliminary data analysis by graphing variables of driving performance over time. Further analyses can be done by utilizing other statistical programs.

Another aspect of the preparation is to test the procedures for playing back a drive in the simulator. This playback function is needed in one of the experiments. The procedures the researchers developed will be used in the planned studies for this work and are as follows. When a participant finishes a drive, an experimenter will transfer the data file generated by the simulator software to then produce a video that is essentially the recording of what the participant saw and did during the drive. The participating subject will then be asked to comment on how and why certain behaviors or decisions were made during the drive. This method will allow the researchers to understand how the participants think of their own driving behaviors (e.g., how fast the subject thought they were driving) and the degree to which this subjective evaluation matches the observed objective behaviors (e.g., the recorded speeds from the speedometer).

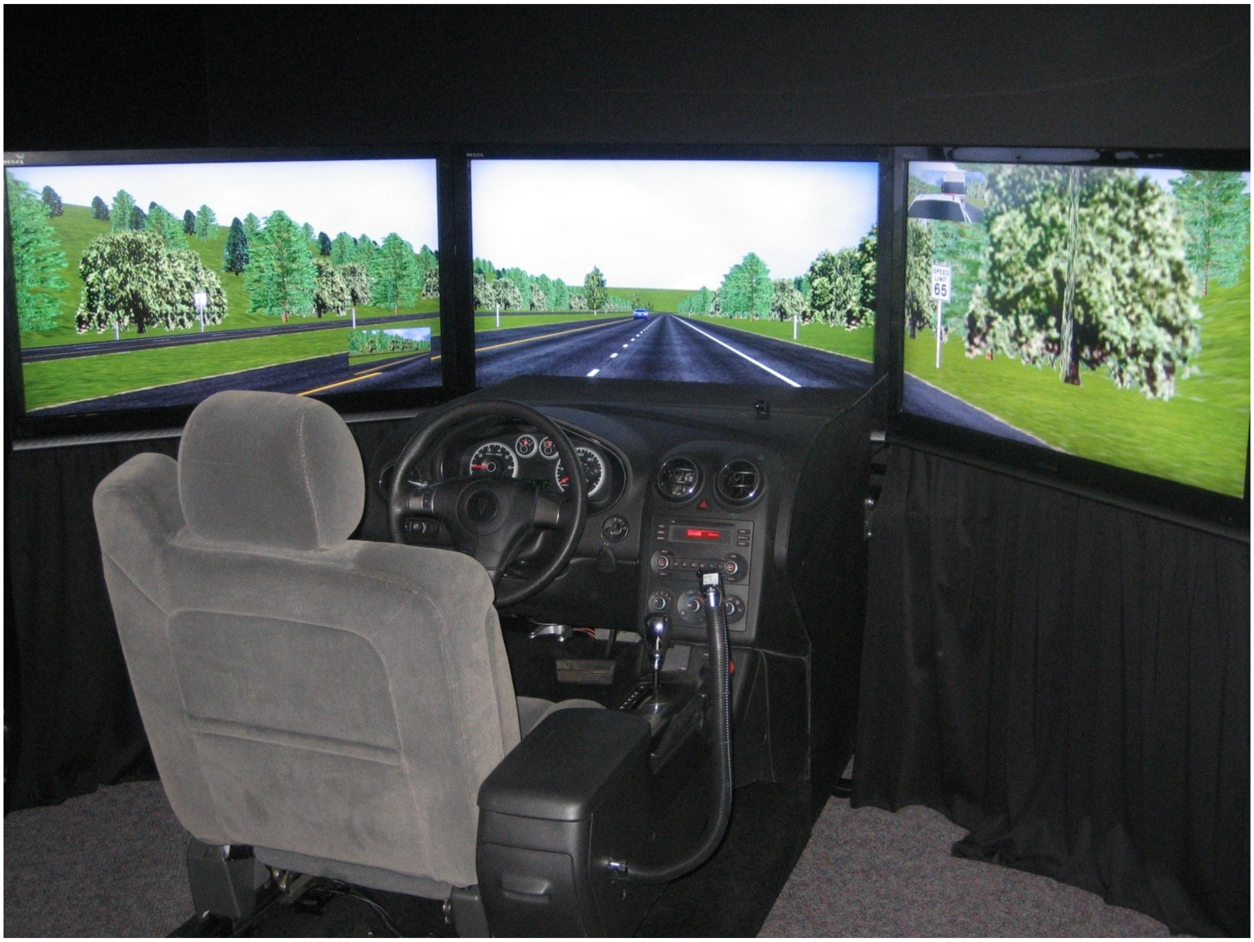
Figure 1. The driving simulator cab and three LCD panels.

4.1.8 Year 2, Milestones 2 & 3: Final Report on developing a protocol for evaluating young driver behavior in the simulator & design of a study to evaluate methods to develop young driver safety interventions

In fulfillment of milestones for Year 2, two protocols were submitted and approved by the Children’s Hospital of Philadelphia’s Institutional Review Board. A synopsis of each protocol is presented below.

4.1.9 Simulated Driving Assessment Protocol: Description and Validation of Its Use as a Multidimensional Driving Simulator Assessment

Protocol Synopsis

Study Title	Validation of a Multidimensional Driving Simulator Assessment
Sponsor	Pennsylvania Department of Health
Study Rationale	Motor vehicle crashes are the leading cause of death and disability in teens and young adults. In order to assess the effectiveness of interventions that target this major public problem, rigorous evaluation methods are needed for outcomes such as driver performance and behavior. On-road evaluations of these outcomes prove difficult for research: they are expensive, time-consuming, introduce additional potential risk, and create challenges for experimental control. Driving simulators are a safer alternative to on-road assessments that are often affordable and provide the needed experimental control for research (Reed & Green, 1999). Though driving simulators would appear to have advantages for evaluating driving performance in teens, the validity of simulator evaluation methods for a multidimensional outcome of driving performance has not been carefully assessed to date.<br><br>In order to use a standardized simulator protocol as a research outcome measure, it is critical to establish its validity as a measure of driving performance. This study will take the first steps toward this goal by examining the validity of a standardized simulator protocol to distinguish between novice and experienced drivers.
Study Objective(s)<br><br>Primary	 <br><br>To evaluate the validity of a driving simulator study protocol in its ability to show significant differences between novice and experienced drivers on driving performance data collected from the simulator and from evaluator scores of the simulated drives.
Secondary	 To further assess the validity of the simulator protocol by examining:<br>The relationship between driving performance data collected from the simulator, evaluator scores of the simulated drives, self-report measures of driving behaviors, and future DMV crash records and citation data<br><br>The differences between learner drivers and newly licensed drivers

	in data collected from the simulator and evaluator scores of the simulated drives.
TEST ARTICLES(s)	This study will include five types of test articles:<br><br>Driving performance data collected from a Realtime Technologies, Inc (Royal Oak, MI) Open Cockpit Research Vehicle Simulator. Realtime Technologies, INC specializes in multi-body vehicle dynamics and graphical simulation and modeling. The simulator is located at the Center for Injury Research and Prevention (CIRP), at the Children's Hospital of Philadelphia<br><br>Several self-report inventories and/or study team administered assessment for both novice and experienced drivers and a parent/legal guardian of novice learner and licensed drivers<br><br>Evaluator assessment of driving performance<br><br>Scanning behavior collected by an Eye Tracker<br><br>PennDOT crash and violation records<br><br>Risk Awareness and Perception Training (RAPT)
Study Design	Cross-sectional study; no interventions are included.
Subject Population<br>Key criteria for inclusion and exclusion:	Inclusion Criteria<br>Three groups of potential participants will be invited to participate:<br><br>Novice male and female licensed drivers<br>Aged 16 through 18.9<br>With a valid PA state driver's license for ≤ 90 days<br>Parent or legal guardian willing to complete surveys about their teen<br><br>Novice male and female learner drivers (Note: This was included in the protocol but not conducted)<br><br>Aged 16 through 18.9<br>With a valid PA state learner's permit<br>Parent or legal guardian willing to complete surveys about their teen<br><br>Experienced male and female drivers<br>Aged 25 through 50<br>With a valid PA state driver's license<br>Licensed for ≥ 5 years<br>Self-reported drives ≥ 100 miles per week<br>No self-reported crashes or moving violations in the past three years

	Exclusion Criteria<br><br>ALL<br><br>Participants with self-reported diagnoses of claustrophobia, migraine headaches, and/or motion sickness (which would preclude ability to use the simulator)<br><br>Females with self-reported pregnancy (a contra-indication to use of a driving simulator)<br><br>Non-English speaking<br><br>FOR EXPERIENCED DRIVERS<br><br>Self-reported crashes or moving violations in the past 3 years
Number of Subjects Overall and at CHOP<br><br>Number of Study Sites	Up to 100 participants, approximately 20 of which will be pilot subjects with non-evaluable data collected and up to 5 subjects who may not complete the protocol.<br><br>CHOP is only study site
Study Duration<br><br>Duration subject participation<br><br>Expected Study Duration	 <br>One study visit for up to 3 hours<br><br>The study duration is anticipated to last up to 12 months
Study Phases<br><br>Recruitment and Potential Subject Identifying<br><br>Screening<br><br>Pre-Simulator Drive Phase	 <br>All Participants will generally be recruited through posted flyers, PA DMV offices, malls, libraries, and by word-of-mouth. In addition, novice teen learner and licensed drivers and their parents may be recruited from a combination of PA DMV licensing centers, CHOP PeRC primary care practice sites, and local public and private high schools in suburban Philadelphia.<br><br>Screening will be conducted over the phone or in person by a research assistant (RA) or research coordinator (RC).<br><br>The pre-drive survey phase of this study will consist of participants and their teen parent / legal guardian completing several self-report

	inventories. Surveys may be completed before, during, or after the study visit.
Simulator Drives	All participants will complete up to 11 simulator drives, including 1 practice drive and up to 10 experimental drives. Driving performance will be evaluated by a) data collected from the driving simulator or b) trained research staff scoring the drives concurrently OR professional driving instructors scoring drives post-study visit via video recordings (by outside consultant).
Post-Simulator Drive Phase	Participants will complete RAPT either at CHOP via computer or remotely via shared desktop. Participants will complete a motion sickness assessment and will be debriefed by study team members.
Driving Record Follow-Up	Participants' PennDOT driving records will be requested and motor vehicle crashes and/or moving violations will be documented. In addition, participants will be contacted to self-report any motor vehicle crashes and/or moving violations.
Safety Evaluations	This is a minimal risk study with no medical procedures or drug interventions.
Statistical and Analytic Plan	Bivariate correlations, multivariate ANOVAs (MANOVAs), and independent t-tests, and linear regression models will be conducted to examine groups' performance on various driving performance data collected from the simulator, the evaluator data from trained evaluators, and self-reported driving behaviors.
Data and Safety Monitoring Plan	The Principal Investigator and lead research coordinator will monitor study procedures to ensure safety and respect for human subjects.

4.1.10 Protocol for Evaluating Internet-Based Interventions with the SDA as an Outcome: Comparison of Driving Performance Among Trained and Untrained Novice Drivers

Protocol Synopsis

Study Title	Comparison of Driving Performance Among Trained and Untrained Novice Drivers: Validation of a Multidimensional Simulated Driving Assessment
Sponsor	Pennsylvania Department of Health
Study Rationale	Motor vehicle crashes are the leading cause of death and disability in teens and young adults. In order to assess the effectiveness of interventions that target this major public problem, rigorous evaluation methods are needed for outcomes such as driver performance and behavior. On-road evaluations of these outcomes prove difficult for research: they are expensive, time-consuming, introduce additional potential risk, and create challenges for experimental control. Driving simulators are a safer alternative to on-road assessments that are often affordable and provide the needed experimental control for research (Reed & Green, 1999). Though driving simulators would appear to have advantages for evaluating driving performance in teens, the validity of simulator evaluation methods for a multidimensional outcome of driving performance has not been carefully assessed to date. It is critical to further examine its validity as a measure of driving performance.<br><br>This study builds on our previous IRB protocol (11-008305) by using a previously-validated hazard perception training program to further examine the validity of a standardized simulator protocol.
Study Objective(s)<br>Primary<br><br><br><br>Secondary	 <br>To examine differences in performance on the simulated driving assessment between novice teen drivers who receive a previously-validated hazard perception training program (RAPT) and novice teen drivers who do not receive the training.<br>To further assess the validity of the simulator protocol by examining the relationship between driving performance data collected from the simulator and eye tracker, self-report measures of driving behaviors, pre-test hazard perception scores, post-test hazard perception scores (for those that receive the training) and future DMV crash records and citation data.
TEST ARTICLES(s)	This study will include five types of test articles:<br><br>Driving performance data collected from a Realtime Technologies, Inc (Royal Oak, MI) Open Cockpit Research Vehicle Simulator. Realtime Technologies, INC specializes in multi-body vehicle

	dynamics and graphical simulation and modeling. The simulator is located at the Center for Injury Research and Prevention (CIRP), at the Children's Hospital of Philadelphia<br><br>Several self-report inventories and/or study team administered assessments for novice drivers.<br><br>Risk Awareness and Perception Training (RAPT) – Assessment: the component of RAPT that is a pre-training assessment tool<br><br>Scanning behavior collected by an eye tracker<br><br>State crash and violation records
Study Design	Prospective study
Subject Population<br>Key criteria for inclusion and exclusion:	Inclusion Criteria<br><br>Novice male and female licensed drivers<br>Aged 16 through 18.9<br>With a valid driver's license for ≤ 180 days<br><br>Exclusion Criteria<br><br>Participants with self-reported diagnoses of claustrophobia, migraine headaches, and/or motion sickness (which would preclude ability to use the simulator)<br><br>Females with self-reported pregnancy (a contra-indication to use of a driving simulator)<br><br>Participants who have previously completed a CHOP driving simulator study<br><br>4. Non-English speaking
Number of Subjects<br><br>Overall and at CHOP<br><br><br>Number of Study Sites	 <br><br>Up to 100 participants, approximately 15 of which will be pilot subjects with non-evaluable data collected and up to 5 subjects who may not complete the protocol.<br><br>CHOP is only study site
Study Duration<br><br>Duration subject	 <br>Pre-visit surveys and up to two study visits:

participation	Trained group: Pre visit surveys and 1) Hazard perception training program (RAPT – pre-training assessment, training program, post-training assessment) visit either via shared desktop or live meeting or a visit to CIRP for completion (up to 1 hour for the pre visit survey and training); and 2) Simulator assessment visit in person at CIRP (up to 3 hours).<br><br>Untrained group: Pre-visit surveys and 1) Simulator assessment visit in person at CIRP (up to 3 hours). Prior to completion of the simulated assessment, the participants in the untrained group will complete the pre-test of hazard perception (RAPT-Assessment).
Expected Study Duration	The study duration is anticipated to last up to 12 months
Study Phases	
Recruitment and Potential Subject Identifying	Recruitment activities may include direct contact with study participants at DMV offices, CHOP PeRC practices, at malls, schools, afterschool activities, libraries, camps, sports activities and through word-of-mouth. Recruitment materials may include: posted flyers, letters, email blasts, and study information sheets.<br><br>All recruitment materials will be submitted to the IRB for review and approval before use in recruitment.
Screening	Screening will be conducted over the phone or in person by a research assistant (RA) or research coordinator (RC). After consent, participants will be randomized to one of two groups: One group will complete the RAPT-training (hazard perception training) and one group will received the RAPT-Assessment
Pre-visit survey data	The pre-drive survey phase of this study will consist of teen participants completing several self-report inventories. Surveys will be completed prior to the simulated driving assessment at CHOP.
Trained Group Visit 1	The group randomized to receive the training will be scheduled for a shared desktop/live meeting or visit to CIRP to complete the RAPT program (pre-training assessment, RAPT training and post-training assessment) .
Trained Group Visit 2	Participants will come to CIRP 1-2 weeks after RAPT training to complete up to 5 simulator drives, including 1 practice drive and up to 4 experimental drives. Driving performance will be evaluated by data collected from the driving simulator and eye tracker .
Untrained Group Visit 1	

Post-Simulator Phase<br><br>Driving Record Follow-Up	The group randomized not to receive the RAPT training will be scheduled for a visit at CIRP to complete up to 5 simulator drives, including 1 practice drive and up to 4 experimental drives. Driving performance will be evaluated by data collected from the driving simulator. Prior to completion of the simulated assessment, the participants in the untrained group will complete the RAPT-Assessment (pre-test of the RAPT hazard perception program).<br><br>All participants will complete a motion sickness assessment and will be debriefed by study team members.<br><br>All participants' driving records will be requested and motor vehicle crashes and/or moving violations will be documented. In addition, participants may be contacted to self-report any motor vehicle crashes and/or moving violations.
Safety Evaluations	This is a minimal risk study with no medical procedures or drug interventions.
Statistical and Analytic Plan	Bivariate correlations, multivariate ANOVAs (MANOVAs), and independent t-tests, and linear regression models will be conducted to examine groups' performance on various driving performance data collected from the simulator, and self-reported driving behaviors.
Data and Safety Monitoring Plan	The Principal Investigator and lead research coordinator will monitor study procedures to ensure safety and respect for human subjects.

4.2 Accomplishments Report for Year 3 Milestones: July 1, 2011 – June 30, 2012

4.2.1 Milestones for Year 3 (July 1, 2011 – June 30, 2012)

**Milestones**	**Project Aims Satisfied**	**Annual Report Submission**
1. Initiate intervention evaluation study	1	7/1/2011-6/30/2012
2. Design study to evaluate marketing/dissemination plan	2	7/1/2011-6/30/2012
3. Complete a report describing an evidence-based framework for the development and evaluation of theoretically-grounded Internet-based interventions to promote safe behaviors among young drivers and their passengers	1	7/1/2011-6/30/2012

4.2.3 Accomplishments for Milestone 1: Initiate intervention evaluation study

The intervention evaluation study involved two protocols: Protocol 1: Validation of the Simulated Driving Assessment (SDA) and Protocol 2: Randomized Trial of the Efficacy of RAPT in Improving Performance on the SDA.

As an initial step in the intervention evaluation study, Protocol 1 was conducted with 15 young, novice drivers (aged 16-18 with no more than 90 days of solo driving experience) and 19 adult experienced drivers (aged 25-50 with at least five years of driving experience) during Year 3. This protocol involved the chosen intervention that met the Framework for the Development of Internet-Based Interventions (Method 1.1), This protocol also further assessed the validity of the Simulated Driving Assessment (developed for Method 1.3). The protocol involved the following steps: (1) Administer pre-drive surveys; (2) train participants in the use of the simulator; (3) instruct participants to complete the simulated –based protocol; (4) administer post-drive surveys, and (5) complete the Risk Awareness and Perception Training (RAPT) program [novice teen drivers only]. Preliminary results suggested that RAPT met content pre-testing standards in that, under direct observation, all young novice driver participants understood the instructions and could complete the training with little help from study staff.

The second protocol was implemented to evaluate RAPT according to the newly developed evaluation framework (Method 1.4 above), via a randomized trial, with the SDA serving as the primary outcome measure. Novice teen drivers were randomized to receive either the RAPT training program [treatment group] or non-training program [control group]. The training group received RAPT and then completed the SDA 1-2 weeks after completing the training. The control group did not receive the RAPT training but completed the SDA and all other study procedures as did the training group.

4.2.4 Accomplishments for Milestone 2: Design study to evaluate marketing/dissemination plan

In Year 3, the research team identified Chris Yang, PhD an Associate Professor of Information Science and Technology in the *I*-School at Drexel University as a local, Pennsylvania-based expert on social media and Web-based information search and retrieval research, expertise that would strongly enhance our work. This new research collaboration between Drs. Winston and Kassam-Adams (from CHOP/Penn) and Yang (from Drexel) established a strong interdisciplinary team (with expertise in pediatrics, information science and technology, behavioral science, engineering, public health) and methods not only for the dissemination research study but also for future translational research in the dissemination of evidence-based interventions for health promotion.

During this year, for Methods 2.1 and 2.2, the team reviewed literature and methods typically applied to the promotion of electronic commerce and adapted them for the promotion of Web-based interventions for teen driver safety. This research resulted in evidence-based strategies for the dissemination of internet-based interventions for teen driver safety (Method 2.1) and the evaluation of these dissemination methods (Method 2.2). The basic principles behind the strategies involve:

(1) Implementing strategies to continuously improve the likelihood that search engines (such as Google) will rank the target Website highly among search results that are presented when a potential user of the intervention (i.e. teen learners or novice drivers and their parents) provides relevant search terms (keywords). [The target Website is the site that presents the evidence-based teen driving intervention.]; and

(2) Implementing strategies to continuously improve awareness of the intervention by Web users who are influential to the target audience (i.e. adults who influence teen learners or novice drivers and their parents) who will then drive traffic to the target Website, and thus to the intervention.

In parallel, Method 2.2 involves monitoring metrics related to evaluating search results, “reach” of messages (through social media) and other strategies to improve awareness of the target Website, and, ultimately, visits to and use of the Website and intervention. Some of these evaluation methods are available commercially; however, the tasks of the research team were to adapt these methods for health and teen driver safety and develop new methods to address the dynamic nature of the Web and social media.

For Method 2.3, the team designed a study to optimize dissemination strategies for TeenDriverSource.org, an evidence-based, Internet-delivered intervention to promote teen

driver safety, based on the team's evidence-based social media strategies. Below is an outline of the objectives, aims, and research methodology for this dissemination study

4.2.5 Dissemination Research Protocol Synopsis

Study Title	Evaluation of Evidenced based methods to disseminate TeenDriverSource.org
Evidence-Based Internet Intervention	TeenDriverSource.org; a resource that is regularly updated with evidence-based information. This Web-based intervention, a resource for multiple stakeholders interested in promoting the safety of teen drivers, was developed based on our systematic best practice framework for the development of evidence-based interventions.<br><br>Note: TeenDriverSource.org is embedded with GoogleAnalytics (www.google.com/analytics) code that enables the capture of aggregated statistics on visitors to the website.
Study Rationale	The National Institutes of Health describes an enormous gap between what we know can improve health and wellness and what is currently being delivered in practice and community settings, and this holds true for preventing the leading cause of teen death, crashes. The Internet holds excellent potential for advancing health and safety as evidenced by the growing number of evidence-based, proven efficacious interventions to improve health and safety. For these interventions to achieve public health impact, however, a systematic, evidence-based marketing and dissemination strategy to reach broad audiences and promote their use. Given the dynamic nature of the Web and social media, a static strategy is not realistic. The strategy must be flexible, monitored and optimized on a continual basis. Therefore, the NIH and NSF have recognized that for e-Health, study designs, including pre-post and adaptive designs, are more efficient and practical than traditional randomized control trials.<br><br>Marketing and dissemination encompass a wide range of potential activities including but not limited to: a) direct to user advertising, b) dissemination and awareness among influencers of the users, c) improved delivery to promote and improve ease in use and d) other methods for optimizing awareness, demand, delivery and use. Support tools, such as electronic newsletters and blogs, can keep stakeholders updated and engaged in promotion of evidence-based interventions and information. The Internet also provides new partners and new dissemination channels for evidence-based intervention delivery (e.g., bloggers as partners and social media networks as dissemination channels) with Twitter and other forms of social media becoming potentially important strategies for health

	information dissemination and communication.
Study Objective	To optimize dissemination strategies for TeenDriverSource.org
AIMS	To test several methods to increase visitors to the internet-based intervention, TeenDriverSource.org.<br>To test several methods to grow the social network of professionals and volunteers interested in receiving evidence-based information about reducing teen driver crashes
STUDY DESIGN	Pre-post study design
METRICS	Primary outcome metrics:<br><br>(Aim 1) Visitors to TeenDriverSource.org): Number of visitors per month to the internet-based intervention, TeenDriverSource.org.<br>(Aim 2) Growth of the social network related to teen driver safety: Number of subscribers to Research-in-Action newsletter and number of followers of SafetyMD on Twitter<br><br>Dissemination method-specific metrics:<br><br>(Method 1) e-Newsletter-specific metrics: Increase in views of newsletter content and sharing of content on social media sites<br><br>(Method 2) SafetyMD Twitter network-specific metrics: Increase in views of teen driver-related content delivered by SafetyMD on Twitter and "retweeting" of this content by followers
STUDY PHASES & METHODS<br><br>Baseline Assessment:	 <br><br>Metrics associated with the primary aims above will be collected as follows:<br><br>Visitors for the teendriversource.org website - from Google Analytics (www.goole.com/analytics)<br><br>Subscribers to the Research-in-Action newsletter – from MailChimp (www.mailchimp.com), the e-newsletter distribution service used for Research-in-Action<br><br>Followers of SafetyMD – from Twitter (www.twitter.com)<br><br>Several months before the initiation of improvement procedures, baseline metrics, both primary outcome metrics (as describe above) and dissemination method-specific metrics (as describe with each method below) will be collected for one month. These metrics will be repeated after each dissemination method and assess the success

	in achieving the goals of achieving the goal of the dissemination method and in increasing the number of visitors to teendriversource.org. (collected for at least one month before initial dissemination method is implemented)
Dissemination Method 1:	The Center for Injury Research and Prevention has delivered the Research-in-Action e-newsletter to a list of approximately 1000 email subscribers. While the newsletter has some success in sharing information about our center's research and interventions, there was little evidence that it increased dissemination of our evidence-based intervention, TeenDriverSource.org. Therefore, this method builds on the established newsletter by improving its design to promote sharing of information on and links to TeenDriverSource.org.<br><br>Initial strategies will be piloted and optimized before final implementation for the study. Examples of strategies to be piloted include: addition of social media functions and channels (including Twitter, Facebook, LinkedIn and Digg) that allow newsletter subscribers to send short messages to their friends on these social media sites about the newsletter or the newsletter stories. For example, after reading a newsletter story, the reader is able to immediately click on the Twitter icon and connect to the Twitter web site on an Internet browser or Twitter app on iPhone/Smart phone to post a tweet and disseminate the short message to her Twitter followers. By using this dissemination method, we extend the utility of the newsletter beyond dissemination through the email subscriber list to also allowing the email recipients to spread the information in the newsletter to their social networks on social media sites. The recipients of these tweets can also further disseminate this content to other followers.<br><br>e-Newsletter-specific metrics: For this method, additional intermediate metrics will assess the effectiveness of the changes as it is related to the e-newsletter.<br><br>Number of views of the newsletter content (overall and by story related to teen driver safety) – from MailChimp<br><br>Number of "shares" of newsletter content (overall and by story content related to teen driver safety and social media channel) – from MailChimp
First Interim Assessment:	Once pilot testing of Dissemination Method 1 is complete and methods are finalized, for one month following the dissemination of the newly enhanced Research-in-Action newsletter, metrics (see above) will be measured, and compared to baseline metrics.

Dissemination Method 2:	The Center also maintains a Twitter presence through SafetyMD. While SafetyMD has more than 1000 followers, its success in ensuring that content is spread further on Twitter is limited, as is its ability to drive traffic to TeenDriverSource.org. With this method, the Twitter social network for SafetyMD will improve the dissemination of teen driver safety information and interventions and Research-in-Action newsletter content to a broader array of people. This enhanced network will include: (1) a broader array of people with interest in road safety, teen health and other communities complimentary to teen driver safety and (2) people who are likely to disseminate information through the social network to more users, producing a magnifier effect.<br><br>Social network analysis with SafetyMD as the primary node will be conducted to determine the followers of SafetyMD with a focus on those who actively retweet messages related to teen driver safety. The Twitter API will be used to develop custom software to identify the network of followers (and their followers) of SafetyMD and the content of their messages. Based on analysis of the network, SafetyMD's network will be expanded to include improved content in messages that will more likely be "retweeted" as well as the identification of new people whom SafetyMD will invite to follow her on Twitter. In addition, SafetyMD followers will be encouraged to subscribe to the Research-in-Action newsletter.<br><br>SafetyMD network-specific metrics: For this method, additional intermediate metrics will assess the effectiveness of the strategies to enhance the SafetyMD network.<br><br>Number of views of SafetyMD tweets and their content (overall and by story related to teen driver safety) – custom software<br><br>Number of retweets of SafetyMD tweets (overall and by story content related to teen driver safety and social media channel)<br>Content of tweets by followers of Safety MD related to teen driver safety – custom software
Final Post-Test Assessment:	Once pilot testing of Dissemination Method 2 is complete and methods are finalized, for one month following enhancements to SafetyMD's social media network on Twitter, metrics (see above) will be measured, and compared to baseline metrics.

4.2.6 Technical Report: An evidence-based framework for the development and evaluation of theoretically-grounded Internet-based interventions to promote safe behaviors among young drivers and their passengers

In fulfillment of milestone #3 for Year 3, the following report was presented for describing an evidence-based framework for the development and evaluation of theoretically-grounded Internet-based interventions to promote safe behaviors among young drivers and their passengers was submitted in Year 3.

Overview

With the increasing advancement in technology and online capabilities, behavioral interventions have radically transformed from in-person programs and interaction into online interactive behavior modification programs (Eng, Gustafson, Henderson, Jimison, & Patrick, 1999; Ritterband, Gonder-Frederick et al., 2003). This explosion of web-based interactive tools to improve health and promote safe behaviors has replaced standard of care in numerous medical and educational settings. Web-based interventions have been developed to treat psychological disorders such as anxiety and depression (Calear, Christensen, Mackinnon, Griffiths, & O'Kearney, 2009; Christensen, Griffiths, Korten, Brittliffe, & Groves, 2004; Christensen, Griffiths, & Jorm, 2004; Christensen, Griffiths, Mackinnon, & Brittliffe, 2006),bulimia nervosa (Pretorious, Rowlands, Ringwood, & Ulrike, 2010), and pediatric Encopresis (Ritterband, Cox et al., 2003), as well as promote safe behaviors (Portnoy, Scott-Sheldon, Johnson, & Carey, 2008).

As essential component in the development and implementation of these web-based interventions in their large scale outcome evaluation, to ensure the developed online tool elicits positive changes in the targeted behavior. Rigorous methodologies have been developed to evaluate non web-based behavioral interventions (Collins, Murphy, Nair, & Strecher, 2005; Dannenberg & Fowler, 1998; Eng et al., 1999; Rounsaville, Carroll, & Onken, 2001). However, little work has focused on systematically establishing rigorous methodologies for evaluating web-based interventions and no research exists on the framework for evaluating web-based applications for safe behaviors among young drivers and their passengers. Eng et al. (1999) established a program evaluation model for evaluating interactive health communication applications. Furthermore, Danaher et al. (2009) reviewed methodological issues in research on web-based behavioral interventions and focused on essential areas of web-based research, including recruitment, user satisfaction, and research design. Furthermore, Winston et al. (2010) developed a practical approach for applying best practices in behavioral interventions to injury prevention.

Based on the work of Eng et al. (1999) and Danaher et al (Danaher & Seeley), as well as recommendations from Winston & Jacobsohn (2010), we will provide a framework for evaluating web-based interventions, specifically focusing on the outcome evaluation of web-based applications for health behavior change. Throughout this framework we will provide an example of applying this guide to the evaluation of an internet-based application for promoting safe behaviors among young drivers and their passengers. This guide can be applied to the development of web-based interventions across topics, including the treatment of psychopathologies and the promotion of healthy behaviors.

The Risk Awareness and Perception Training (RAPT) program was designed to teach novice drivers about risky situations normally encountering during driving(Fisher, Pollatsek,

& Pradhan, 2006; Pradhan, Fisher, & Pollatsek, 2006). We will reference RAPT throughout this framework for the outcome evaluation of web-based interventions.
While implementing several key evaluation steps and activates, we suggest abiding by the key principles for evaluation, as recommended by the Science Panel on Interactive Communication and Health (ICH):

Table 1. Key principles for Evaluation of Interactive Communication and Health (ICH)

Guideline	Key Components	Questions to Ask
Evaluation Should be practical	Methods should reflect real-world considerations	What is the problem that needs to be addressed? Will persons with limited resources, experienced or training in evaluation methodologies able to participate fully in the evaluation process?
Evaluation should be proactive	Evaluation should seek to prevent problems and help create high-quality products	What the is key outcome of the intervention?
Evaluation should have a clear purpose	A clear vision of how results will be used to improve the design, implementation, or use of the application	How will results be used to improve the design, implementation, or use of the application?
Evaluation should be shared responsibly	Responsibly of the intervention should be shared across developers, providers, purchasers, consumers and policy makers	Do all members of the team have direct responsibility to the implementation of the intervention?
Evaluation should be ubiquitous in product development	Evaluation methods should be woven throughout the entire design, implementation, and dissemination phases of product development	Does my methodology follow consistency throughout the entire process of development, testing, and disseminating the intervention?

Source: Eng, T. R., Gustafson, D. H., Henderson, J., Jimison, H., & Patrick, K. (1999). Introduction to evaluation of interactive health communication applications.American Journal of Preventive Medicine, 16, 10-15
Evaluation Framework
To begin developing our methodological framework for evaluating web-based interventions for safe behaviors among young drivers and their passengers, we have outlined the phases of web-based intervention evaluation into three phases:
Phase I: Formative Evaluation
Before implementing an outcome evaluation of the web-based intervention, formative evaluation is essential to assess the nature of the problem and needs of the target population(s) (Eng et al., 1999). Formative evaluation activities revolve around web-based intervention conceptualization and design (Eng et al., 1999). See Kohser (2011) report for framework on pre-testing content of intervention-based interventions.

Phase II: Process Evaluation
Process evaluation assesses the feasibility of the implantation of the web-based application. It monitors operational characteristics of the intervention and assesses logistical concerns such as security, accuracy, reliability, usability, and response time (Eng et al., 1999). See Kohser (2011) report for framework on pre-testing content of intervention-based interventions.
Phase III: Outcome Evaluation
Once a process evaluation has been completed, the next step in the evaluation process of a web-based intervention is to employ and implement an experimental design to evaluate the efficacy of the online behavioral intervention. Outcome evaluation addresses assessment and necessary refinement of the intervention, prior to moving into large-scale effectiveness evaluation (dissemination). We have outlined eight important steps to outcome evaluation:
Step 1: Define Research Questions
Define research questions that clearly reflect the web-based intervention's intention and purpose.
Example: The goal of RAPT is to improve scanning behavior among novice teen drivers. Teaching novice drivers to scan and anticipate potential hazards significantly decreases the risk of crashing. Thus, a potential research question may be:
"Do young drivers who are trained to be more aware of hidden risks in a PC-based training program drive more cautiously in a simulator than untrained novice drivers (Fisher et al., 2002)?"
Step 2: Select Key Outcomes
They key outcome is the desired result which the intervention seeks to effect. This outcome should be a clear, measurable, and long-term outcome related to the purpose of the intervention (Winston & Jacobsohn, 2010)
Example: Measure crash outcome after being trained on RAPT would not be the most suitable and practical tool for measure the outcome of an intervention. Thus, we suggest focusing on practical and short-outcomes related to the purpose of the intervention. For example, in completing a post-training simulated drive did novice trained drivers:
Perform better than novice untrained drivers?
Remain more cautious than untrained drivers, even with no visible hazard?
Able to identify more hazards than untrained novice drivers?
STEP 3: SELECT ASSESSMENT TOOLS AND INSTRUMENTS
Select assessment methods that most accurately measure the target construct(s). Methods of assessment tools may include self-report surveys, interviews, and assessments. Whenever possible, triangulation should be implemented.
Example: The evaluation of RAPT utilizes an eye tracker to assess the degree to which novice teen drivers' scan for potential dangers on the road. In addition, driving simulators can be utilized to measure the efficacy of an intervention as safer alternative on-road assessments.
STEP 4: DESIGN CONSIDERATION
Experimental Design
Choose and implement a research design to evaluate the short and long-term impact of the behavioral intervention. Danaher et al (2009) outlines several types of studies designs utilized in evaluation studies of internet-based interventions.
Blended Designs

A blended design contains essential features of efficacy research, including randomization, use of control conditions, independent assessment of outcome, and monitoring of treatment delivery. However, this design also expands to research questions on the effectiveness (or external validity) of the intervention. Specific examples of blended designs include practical clinical trials (PCT) and pragmatic randomized controlled trials (P-RCT).
Example: Design a study to evaluate both the immediate outcomes of implementing RAPT (i.e. performance on a driving simulator) and long-term outcome of RAPT (i.e. does it prevent motor vehicle crashes).
Adjunctive Designs
Adjunctive designs include combine web-based interventions with other types of treatments.
Example: An evaluation of a web-based intervention for chronic disease self-management utilized the online tool as a main program following completion of a clinic-based program. An adjunctive design would be useful for a web-based application to assist teens during the learning to drive phase, which also includes an in-vehicle or classroom component.
Dismantling and Mantling Designs
The goal of these designs is to isolate the effect of a specific web-based program component(s).Mantling designs are used to determine the effect components or features during the early stage of development, while dismantling design are utilized after the application has been proven effective (Danaher & Seeley, 2009).
Example: This design could study and asses the feasibility of including various environments within RAPT, by examining individual components, events, or scenarios.
Comparison Group Consideration
Choose the type of comparison or control conditions used in experimental trials. Three comparison conditions have been utilized and widely discussed in the evaluation of web-based interventions.
Clinic or Standard/Usual Care Comparison Condition
Standard of care designs agree how a relatively newer web-based treatment compares to a more traditional treatment approach. Advantages of this type of design include addressing a) ethical concerns about providing treatment to all participants and b) practical issues around recruitment.
Example: Comparing a newly developed web-based intervention to promote safe driving behavior in novice teen drivers to the traditional 5-hour classroom training.
Web Comparison Group
Using web comparison groups can control for demand characteristics and participant expectancies, as well as have many practical benefits revolved around implementation and broad recruitment to various diverse populations. A basic information website control condition is presented facts about the target behavior and possible treatment recommendations. An enhanced condition usually contains tailored content which creates an individualized behavioral plan through various online features.
Example: An online application designed to assist and guide teens through the learning to drive phase, the web comparison group may either be presented with basic information and recommendations on how to learn to drive or may be given a behavioral plan to focus on specific areas of driving that the participant struggles with (i.e., environment, maneuver).
No-Treatment and Waiting List Controls
Waitlist / no-treatment controls offer practical advantages to the researcher and control for the passage of time. However, it provides a limited value in its ability to provide further

explanation or magnitude of a given process or witness behavior change. This study design is typically used during formative evaluation stages of intervention development.

STEP 5: DEVELOP RECRUITMENT PLAN

Determine the most efficient recruitment methods to recruit participants that meet the target population. As in any area of behavioral research, it is essential to avoid scenarios in which too few participants are enrolled (Danaher & Seeley, 2009). Recruitment focuses on 1) how participants are recruited and 2) the location where participants are recruited. Open recruitment methods are not recommended, as it is difficult to enroll participants that meet inclusion and exclusion criteria (Danaher & Seeley, 2009). Danaher et al (2009) recommend using a limited recruitment approach, by focuses recruitment on members of the targeted population.

Example: To measure the effectiveness of this training tool for novice drivers, Fisher et al. (2006) recruit young novice drivers through local area driving schools (Pollatsek, Narayanaan, Pradhan, & Fisher, 2006; Pradhan et al., 2006) and high schools (Pollatsek et al., 2006). These sites were chosen to best recruit the target population.

STEP 6: DESIGN STATISTICAL ANALYSES PLAN

Develop and implement a statistical analyses plan to accurately measure whether the hypothesized behavior change occurred.

Example: Evaluation of RAPT involves a pre/post design, where subjects are administered an assessment (i.e. simulator assessment) before and after completing the PC-based training program. An analyses plan designed to investigate significant differences across time (repeated measures) should be utilized.

STEP 7: REFINE INTERVENTIONS AND BEHAVIOR CHANGE MODEL W/ KNOWLEDGE GAINED FROM INTERVENTION EVALUATION

If outcome evaluation proves unsuccessful, make necessary revisions to the behavior change model, the program theory, and/or the intervention content (Winston & Jacobsohn, 2010). Only continue to the next step (Step 8) if intervention is proven successful.

Example: If RAPT is not shown to be effective in teaching novice teen drivers to scan for potential hazards on the road, refine the interventions to closely match the behavior change model.

STEP 8: DEVELOP LARGE-SCALE DISSEMINATION EVALUATION

Conduct a large-scale effectiveness evaluation to address issues of generalizability, implementation, cost-effectiveness, and social validity (Danaher & Seeley, 2009).

Table 1. Recommended steps for outcome evaluation of web-based interventions

Systematic Framework for Outcome Evaluation of Web-Based Intervention	
Step 1	Define Research Questions
Step 2	Select Key Outcomes
Step 3	Select Assessment Tools and Instruments
Step 4	Design Consideration<br>Experimental Design<br>Comparison Group Consideration
Step 5	Develop Recruitment Plan
Step 6	Develop Statistical Analysis Plan
Step 7	Refine Interventions and Behavior Change Model
Step 8	Design Dissemination Plan

References

Calear, A. L., Christensen, H., Mackinnon, A., Griffiths, K. M., & O'Kearney, R. (2009). The YouthMood Project: A cluster randomized controlled trial of an online cognitive behavioral program with adolescents. Journal of Consulting and Clinical Psychology, 77, 1021-1032. 10.1037/a0017391

Christensen, H., Griffiths, K., Korten, A., Brittliffe, K., & Groves, C. (2004). A comparison of changes in anxiety and depression symptoms of spontaneous users and trial participants of a cognitive behavior therapy website. JOURNAL OF MEDICAL INTERNET RESEARCH, 6

Christensen, H., Griffiths, K. M., & Jorm, A. F. (2004). Delivering interventions for depression by using the internet: randomised controlled trial. [Article]. BMJ: British Medical Journal, 328, 265-268

Christensen, H., Griffiths, K. M., Mackinnon, A. J., & Brittliffe, K. (2006). Online randomized controlled trial of brief and full cognitive behaviour therapy for depression. Psychological Medicine: A Journal of Research in Psychiatry and the Allied Sciences, 36, 1737-1746. 10.1017/s0033291706008695

Collins, L., Murphy, S., Nair, V., & Strecher, V. (2005). A strategy for optimizing and evaluating behavioral interventions. Annals of Behavioral Medicine, 30, 65-73. 10.1207/s15324796abm3001_8

Danaher, B., & Seeley, J. (2009). Methodological Issues in Research on Web-Based Behavioral Interventions. Annals of Behavioral Medicine, 38, 28-39. 10.1007/s12160-009-9129-0

Dannenberg, A. L., & Fowler, C. J. (1998). Evaluation of interventions to prevent injuries: an overview. Inj Prev, 4, 141-147

Eng, T. R., Gustafson, D. H., Henderson, J., Jimison, H., & Patrick, K. (1999). Introduction to evaluation of interactive health communication applications. [doi: DOI: 10.1016/S0749-3797(98)00107-X]. American Journal of Preventive Medicine, 16, 10-15

Fisher, D. L., Laurie, N. E., Glaser, R., Connerney, K., Pollatsek, A., Duffy, S. A. (2002). Use of a Fixed-Base Driving Simulator to Evaluate the Effects of Experience and PC-Based Risk Awareness Training on Drivers' Decisions. Human Factors: The Journal of the Human Factors and Ergonomics Society, 44, 287-302. 10.1518/0018720024497853

Fisher, D. L., Pollatsek, A. P., & Pradhan, A. (2006). Can novice drivers be trained to scan for information that will reduce their likelihood of a crash? [Article]. Injury Prevention, 12, i25-i29. 10.1136/ip.2006.012021
Pollatsek, A., Narayanaan, V., Pradhan, A., & Fisher, D. L. (2006). Using Eye Movements to Evaluate a PC-Based Risk Awareness and Perception Training Program on a Driving Simulator. Human Factors: The Journal of the Human Factors and Ergonomics Society, 48, 447-464
Portnoy, D. B., Scott-Sheldon, L. A. J., Johnson, B. T., & Carey, M. P. (2008). Computer-delivered interventions for health promotion and behavioral risk reduction: A meta-analysis of 75 randomized controlled trials, 1988-2007. [doi: DOI: 10.1016/j.ypmed.2008.02.014]. Preventive Medicine, 47, 3-16
Pradhan, A. K., Fisher, D. L., & Pollatsek, A. (2006). Risk perception training for novice drivers - Evaluating duration of effects of training on a driving simulator Highway Safety: Law Enforcement; Alcohol; Driver Training; Safety Planning and Management; Commercial Vehicles; and Motorcycles (pp. 58-64).
Pretorious, N., Rowlands, L., Ringwood, S., & Ulrike, S. (2010). Young peoples perceptions of and reasons for accessing a webbased cognitive behavioural intervention for bulimia nervosa. European Eating Disorders Review: The Journal of the Eating Disorders Association, 18, 197-206
Ritterband, L. M., Cox, D. C., Walker, L., Kovatchev, B., McKnight, L., Patel, K. (2003). An Internet Intervention as Adjunctive Therapy for Pediatric Encopresis. Journal of Consulting and Clinical Psychology, 71, 910-917
Ritterband, L. M., Gonder-Frederick, L. A., Cox, D. J., Clifton, A. D., West, R. W., & Borowitz, S. M. (2003). Internet interventions: In review, in use, and into the future. Professional Psychology: Research and Practice, 34, 527-534. 10.1037/0735-7028.34.5.527
Rounsaville, B. J., Carroll, K. M., & Onken, L. S. (2001). A Stage Model of Behavioral Therapies Research: Getting Started and Moving on From Stage I. Clinical Psychology: Science and Practice, 8, 133-142. 10.1093/clipsy.8.2.133
Winston, F. K., & Jacobsohn, L. (2010). A practical approach for applying best practices in behavioural interventions to injury prevention. Injury Prevention, 16, 107-112. 10.1136/ip.2009.021972

4.3 Accomplishments Report for Year 4 Milestones: July 1, 2012 – June 30, 2013

4.3.1 Milestones for Year 4 (July 1, 2012 – June 30, 2013)

**Milestones**	**Project Aims Satisfied**	**Annual Report Submission**
1. Complete the intervention evaluation study and analyze the results	1	7/1/2012-6/30/2013
2. Conduct the marketing/dissemination study	2	7/1/2012-6/30/2013
3. Complete a report describing an evidence-based framework for the development and evaluation of strategies to market/disseminate Internet-based interventions to promote safe behaviors among young drivers and their passengers	2	7/1/2012-6/30/2013

4.3.2 Complete the intervention evaluation study and analyze the results (Year 4, Milestone 1)

4.3.3 Development of the Simulated Driving Assessment (SDA) Protocol

Note: We extended the scope of work of Year 2, Milestone 2 because no validated assessment of young driver performance was found. This exciting line of work resulted in a very strong and robust Simulated Driving Assessment. The extension of this milestone allowed us the capability to incorporate the SDA into future studies.

In order to develop a protocol for evaluating young driver behavior in the simulator, we used an analysis of existing teen crash data from the National Motor Vehicle Crash Causation Survey, integrative literature review and expert opinion. There are two main outcomes for the development of this protocol:1) a common set of driver performance measures for scoring; and 2) the set of evidence-based simulated scenarios in the protocol.

First, for the common set of driver performance measures, we reviewed the literature on the known driving performance deficits of novice teen drivers, examined scoring mechanisms of critical driving performance metrics in the scoring of simulated drives and on-road evaluations, evaluated our findings with experts in the field for an iterative process of review

and feedback. We outlined eight critical domains of driving: 1). Speed management; 2). Road position; 3). Gap selection; 4). Managing a blind spot; 5). Hazard anticipation and response; 6). Attention maintenance; 7). Communication and right of way, and 8). Vehicle control. In the eight domains, we determined 12 performance metrics that could be scored.

Second, for the development of the simulator scenarios, we relied on a comprehensive study of serious crashes: the National Motor Vehicle Crash Causation Survey (NMVCCS). NMVCCS systematically collected on-scene crash investigation data on vehicles, roadways, environmental conditions and human behavioral factors likely to contribute to a crash. We analyzed a subsample of nationally representative crashes involving 16-18 year olds who were driving alone or accompanied by peer passengers. The four most frequent (weighted) crash configurations in NMVCCS for this subsample included turning left at an intersection, right roadside departure, left roadside departure, and rear-end collisions. We chose the turning left at an intersection, right roadside departure, and rear-end collisions for the development of our simulated scenarios.

The Simulated Driving Assessment (SDA) is comprised of the common set of performance measures and simulated scenarios based on the most frequent crash configurations. The SDA lasts approximately 35-40 minutes and includes an unscored familiarization segment (~5-10 min) followed by three scored modules, each lasting ~10 minutes. During the SDA scored modules, participants were exposed to variations of the NMVCCS-derived most common teen driver crash configurations (rear-end collisions, left turn intersections collisions, and right side run-off the road events), distributed across three 10-minute drive modules. If the participant drove in a safe manner, the crashes could be avoided. Between the potential crash scenarios, the SDA included stretches of straight road, curves and turns that were not intended to be potential crash scenarios, rather to expose participants to naturally occurring driving environments.

4.3.4 Validation of the Simulated Driving Assessment (SDA)

Defining constructs - Experience

To evaluate the validity of the SDA, We enrolled two groups: an “inexperienced” group aged 16-17 years who received a Pennsylvania (PA) provisional license 90 days or fewer prior to study enrollment, and an “experienced” group aged 25-50 years with a valid PA license for more than 5 years, driving at least 100 miles per week and no collisions or moving violations in the past 3 years. Individuals were excluded for a self-reported history of migraines, motion sickness, pregnancy or if they were non-English speaking. Inexperienced participants were recruited via mailings from pediatric primary care facilities, driving schools, and word of mouth. Experienced participants were recruited via flyers, the Wharton Behavioral Lab at the University of Pennsylvania, and word of mouth.

Defining constructs – Safe Driving skill

A professional driving evaluator (certified to teach driving and administer on-road licensing tests in Pennsylvania) rated participants’ safe driving skill based on review of video

recordings of participant performance for the three drive modules (not including the familiarization segment).[20] The driving evaluator viewed video of the participant’s performance in the SDA through four-quadrant divided screen (Figure 1 below).

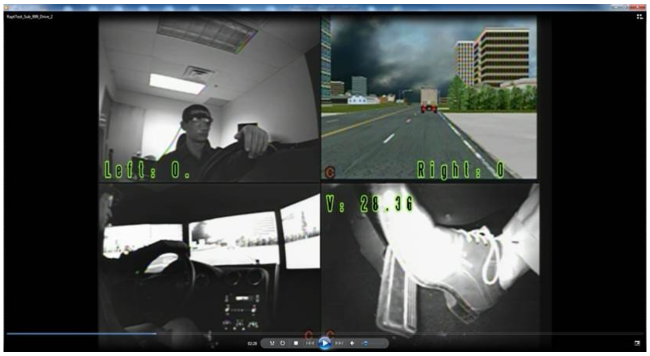

Figure 1. Four quadrant screen used for rating of participant’s driving skill.

One quadrant displayed the center channel of the simulator, or the center simulator monitor depicting the forward roadway that the participant viewed; the second quadrant displayed video from the camera situated to the left of the brake pedal, allowing for view of the brake and throttle behavior; the third quadrant displayed video from the camera over the participant’s right shoulder; and fourth quadrant displayed video from the camera pointed towards the participant’s face. Speed in mph and left and right turn signals were also displayed on the quadrants. The evaluator was blinded to participant driving experience, and the evaluator did not have access to the simulator-derived metrics (except turn signal and speed displayed on video). The evaluator was instructed to review the entire video footage of each participant’s scored drives (including all segments) to produce an overall assessment of each driver’s safe driving skill. A team of four driving experts defined eight domains of driving that were used in evaluator’s skill assessment.[20] The eight domains included (1) speed management, (2) road positioning, (3) gap selection, (4) managing blind spot, (5) hazard anticipation and response, (6) attention maintenance, (7) communication and right of way, and (8) vehicle control. The evaluator was instructed to rate each participant for each domain on a scale of 0-10 for driving skill in comparison to all drivers, regardless of age and experience for each of the domains. Based on the assumption that an average score of 7 or higher across all domains demonstrates safe driving skill, a total score of 56 across all 8 domains was set as the cutoff between skilled and less skilled drivers. Strong internal consistency was found among the 8 driving domains (Cronbach’s alpha=.97) and a significant bivariate relationship between the overall skill rating assessment and the skilled/less skilled derived variable ($p<.01$).[21]

Based on the driving evaluator’s rating for derived safe driving skill (domain score cutoff =56), our sample consisted of 39.5% (n=15) skilled drivers. The skilled drivers represented 43% of all inexperienced drivers and 35% of all experienced drivers ($p=0.74$).[21] Previous results examined the relationship between the driving evaluator’s ratings and the presence of police-reported collisions as a proxy for poor driving skill.[21] Of the 8 experienced drivers (87.5%) who had at least one police-reported crash over their entire licensure in

Pennsylvania, seven were categorized as less skilled, resulting in a sensitivity of 0.87 (95% CI: 0.47-0.99) in the driving evaluator ratings in detecting those who had police-reported crash histories. The only participant categorized as skilled who also had a police-reported crash scored only slightly above the derived cutoff value of 56 (composite score=57). Five of the 9 participants with no police-reported collisions were rated as skilled resulting in a specificity of 0.56 (95% CI: 0.23-0.85).

All study procedures were approved by the Institutional Review Board at the Children's Hospital of Philadelphia.

4.3.5 Summary of Findings on the Validation of the new Simulated Driving Assessment

We found evidence that the SDA was a valid stimulus to elicit difference in performance among novice teen and experienced adult drivers, as well as driving skill. Table 1 below lists all of the submitted/accepted manuscripts that describe the validity of the SDA to differentiate driving performance.

Table 1. List of submitted manuscripts describing the construct validity of the SDA.

Title	Year	Journal	Synopsis	Status
Headway time and crashes among novice teens and experienced adult drivers in a simulated lead truck braking scenario	2013	Proceedings of the 7th International Driving Symposium on Human Factors in Driver Assessment, Training and Vehicle Design	In one of our lead truck braking events in the SDA, we found that teens crashed more than adults, and teens had a shorter headway time than adults	Accepted
Headway time errors, safe driving skill, and experience: An initial validation of the Simulated Driving Assessment	2014	93rd Annual Meeting of the Transportation Research Board (TRB) Compendium of Papers	Participants categorized as less skilled by a professional driving evaluator had more headway time errors than those who were categorized as skilled (controlling for experience). Inexperienced participants had more headway	Accepted

			time errors than experienced participants (controlling for safe driving skill)	
Experience and skill predict failure to brake errors: Further validation of the simulated driving assessment	2014	SAE 2014 World Congress & Exhibition Compendium of Papers	Participants categorized as less skilled by a professional driving evaluator had more failure to brake errors than those who were categorized as skilled (controlling for experience). Inexperienced participants had more failure to brake errors than experienced participants (controlling for safe driving skill)	Accepted

In one of our lead truck braking events in the SDA, we found that teens crashed more than adults, and teens had a shorter headway time than adults. [22] Further, across all rear-end events, both experience and safe driving skill were found to be independent predictors of total headway time errors and failure to brake errors. Participants categorized as less skilled by a professional driving evaluator had more headway time errors and failure to brake errors than those who were categorized as skilled (controlling for experience). Inexperienced participants had more headway time errors and failure to brake errors than experienced participants (controlling for safe driving skill). [23]

4.3.6 Randomized Trial of the Efficacy of Internet-based Risk Awareness and Perception Training (RAPT) in Improving Performance on the Simulated Driving Assessment (SDA) (Year 4, Milestone 1)

4.3.7 Summary of methods for randomized trial of RAPT with SDA as the validated outcome

To evaluate a web-based intervention for young driver safety, we used a randomized controlled trial design and enrolled novice teen drivers and used the SDA as our outcome

measure. Participants were randomized to 1) an intervention group that received RAPT (Risk Awareness and Perception Training) (Trained); or 2) a control group that received no training (Untrained). The RAPT web-based intervention developed by the University of Massachusetts-Amherst involved a baseline assessment, training tutorial, and post-assessment component.

During the baseline assessment of RAPT, teens were required to detect potential hazards on the screen by clicking their mouse on where they thought a potential hazard occurred. Each assessment included nine scenarios for which a point was awarded for each scenario when a correct detection occurred. The points were summed across all nine scenarios with a potential score range of 0-9 for each assessment. Both Untrained and Trained teens completed the baseline assessment. Then, only the Trained teens completed the RAPT training and RAPT post-assessment. The training component involved teaching participants to anticipate hazards in different scenarios. Teens completed the SDA protocol two weeks after 1) baseline assessment, for the Untrained group; or 2) training, for the Trained group .

4.3.8 Summary of findings from the randomized trial of RAPT

A total of 19 Untrained and 18 Trained teens had complete data for RAPT hazard anticipation scores and from the SDA. Demonstrating comparability between the Untrained and Trained groups, there was no statistically significant difference ($p=0.73$) between the baseline RAPT hazard anticipation scores for the Untrained (median=5; IQR: 4-6) and Trained (median=5; IQR: 4-5) teens. Demonstrating that the content of RAPT produced the desired training, within the Trained teens, there was statistically significant difference ($p<0.0001$) between baseline (median=5; IQR: 4-5) and post (median=8; IQR=0) RAPT hazard anticipation scores. Also, a statistically significant difference ($p<0.0001$) was observed when comparing the baseline RAPT score for Untrained (median=5; IQR: 4-6) and the post-RAPT score for Trained (median=8; IQR=0) teens. These results suggest that the two groups were comparable regarding risk awareness and perception of hazards at baseline: RAPT-hazard anticipation skills were comparable; and as hypothesized based on previous results and the theoretical grounding of the intervention, the RAPT intervention produced a training effect as evidenced by significantly improved hazard anticipation scores for the Trained teens.

However, utilizing the rigorous evaluation methodology developed in this project allowed a more extensive examination of the potential real-world impact of the intervention. The new validated outcome measure of driving performance, the SDA, revealed a crucial gap in translating hazard anticipation skills to performance in complex driving situations. The SDA results indicated no difference in simulated driving performance. Crashes were summed across all of the 22 blocks in the three experimental drives, and the proportion of teens having at least one crash during the entire SDA for the Untrained and Trained teens was 47% and 50% respectively ($p=0.87$). There was no statistically significant difference ($p=0.50$) in the distribution of total crashes for the Untrained (median=0; IQR: 0-1) and Trained (median=0.5; IQR: 0-2) teens.

This methodologically sound evaluation of driving performance demonstrated the value of the RAPT intervention and potential areas for its improvement in crash prevention efforts. The teens in the Trained group learned skills related to hazard anticipation, as evidenced by the improved scores post-RAPT training. However, the results from the SDA indicated that the teens did not necessarily employ these skills in high risk, complex driving scenarios – scenarios that commonly result in young driver crashes. There was a gap between knowledge gained and translation to performance that prevents crashes. We discussed our findings with the lead investigator on RAPT and strategized ways to improve hazard anticipation training programs for novice teen drivers, including incorporating additional elements for the intervention that have been shown by others to improve actual driving performance, including commentary driving.

Crash avoidance requires situation awareness. The safe, skilled driver perceives her driving environment through relentless, focused scanning and notices the driver on her right is about to make a move. She comprehends this as a potential hazard and shifts attention to the most relevant elements of the road – the immediate space cushion around her car. She predicts that the driver may cut her off and acts quickly. She releases the throttle (after checking her rear-view mirror) and, if necessary, brakes with sufficient pressure to try to regain a three-second following distance. When she sees the other car's brake lights, she is ready, calmly but assuredly braking to avoid crashing. She is showing "situation awareness," a much-needed skill for safe driving.

RAPT resulted in improved scanning or perception of her environment but did not necessarily result in comprehension, prediction and action. The PA DOH grant allowed our team to develop a rigorous, standardized, valid assessment of driving performance that has implications beyond evaluation of hazard anticipation training.

4.3.8 Conduct the marketing/dissemination study and analyze the results (Year 4, Milestone 2)

For ease of reading, the description and our results of the marketing and dissemination study are presented below in Year 5 Milestones (*Analyze results of marketing/dissemination study).*

4.3.9 Technical reports describing an evidence-based framework for the development and evaluation of strategies to market/disseminate Internet-based interventions to promote safe behaviors among young drivers and their passengers (Year 4, Milestone 3)

In fulfillment of milestone # 3 from Year 4, two technical reports were submitted in Year 4 that described an evidence-based framework for the development and evaluation of strategies to market/disseminate Internet-based interventions to promote safe behaviors among young drivers and their passengers. Both reports are presented below:

4.3.10 Technical Report: Marketing and dissemination for Internet-based interventions

Overview
The internet has become a favored source for health-related information (Morahan-Martin, 2004). While many people search the Web for health information, less than 40% of the available health information found online is accurate (Scullard, Peacock, & Davies, 2010). Giving the amount of misinformation available, when developing high quality Internet-based resources or interventions for health information, it is vitally important to plan for the dissemination of these resources Without a solid dissemination plan interventions cannot achieve their intended goals (such as increasing safety behaviors to save people's lives) (Sogolow, Sleet, & Saul, 2007).

To date, best practices for how to disseminate quality web-based interventions have yet to be established (Crutzen et al., 2008). Over the past decade, three distinct methods of dissemination/marketing via the internet have emerged: Search Engine Optimization (Section 1.0); Paid Online Advertising (Section 2.0), and Participating in Social Media (Section 3.0). These three methods can be very powerful, resource-friendly tools when used together strategically. An example of applying these three methods together to promote a safe driving internet intervention is provided in Section 4.0. Section 5.0 contains additional resources.

1.0 Search Engine Optimization
According to Pew Internet, in 2010 77% of adults utilized the internet. Of those, some 87% of used a search engine to find information online, 84% have searched for health-related information, and 86% used social networking sites (Pew Internet & American Life Project, 2010). For teenagers, aged 12-17, the number of those that utilize the internet increases to 93%, with 73% using social networking sites (Pew Internet & American Life Project, 2009).

Search Engines, such as Google and Yahoo, use a sophisticated formula to determine the order in which websites are listed in any given search. Search Engine Optimization (SEO) is defined as tuning the website to capitalize on these formulas such that the website appears for users during searches (Maloney, 2005). SEO is fairly simple for those sites with uncommon search terms, but provides a challenge for those site requiring more common, competitive search terms. To achieve SEO, select keywords, place keywords throughout the website, and use a linking campaign.

1.1 <u>Selecting Search Terms or Keywords</u>
Selecting the relevant search terms/keywords that people would use to find the targeted internet intervention is the foundation of search engine optimization. When selecting key words, consider words someone from the target audience would when searching for information on the topic.

Example for Internet-based Intervention aimed at Teen Drivers
- Target audience: Teenagers learning to drive or their parents
- Expert keyword: Safe Driving Behaviors
- Teenager or Parent keyword: Driver's Ed, teaching driving, learning how to drive

1.2 Placing Search Terms throughout your site

Once a list of search terms/keywords has been developed, use this list to optimize the site. Include these search terms within the metadata and body of the website. As for body content, search engines focus on text. Some experts claim that the higher up on the page the better, theory being that search engines “spider through” only a certain amount of text on each page (Maloney, 2005).

The most powerful place to insert metadata is in the page title. Other places you can include search terms within metadata are within the page descriptions or images.

To insert search terms into metadata, view the source code:

- For the page title: <title>Page Title </title>.
- For the page description: <<meta description="Page Description"/>
- For the images:

1.3 Linking

Another way that search engines rank websites for SEO is by the number of quality websites that link to the site. The higher the number of links to the site the higher the site will be ranked (Maloney, 2005). To increase the number of quality sites that link to the Internet Intervention, create a linking campaign: Identify sites that are reputable and relevant to the Intervention and request they link to you.

2.0 Paid Online Advertising

In comparison to a traditional marketing campaign, online advertising can be strikingly inexpensive. There are many ways to advertise over the internet including buying advertising on any of the search engines, on social networking sites, or banner ads on targeted websites that accept advertising dollars (for example:WebMD).

2.1 Search Engine Advertising

Of the top five search engines, Google is by far the most used. In North America, from May 2010 to April 2011, Google’s share of the usage market hovered near 90%, while the remaining four – Yahoo, bing, AOL, and Ask Jeeves – all hovered under 10% (StatsCounter Global Stats, 2011). For that reason, this report focuses on Google Adwords; however, most paid advertizing mechanisms work in a similar fashion (Center for Disease Control and Prevention, 2007).

It is important to note that internet users tend to view sponsored links critically. One study found that 77% of participants favored organic over sponsored, however, the Title of the ad was what was used to determine relevance. If the Title was found to be relevant then the user was more likely to move on to the summary (Jansen & Resnick, 2006). If the intent of the user is informational, then they are likely to click on the organic listing 84% of the time and the sponsored link 16% of the time (Jansen & Spink, 2009).

Taking this knowledge into account and developing a well thought-out ad campaign, Google Adwords (Adwords) can be a great tool in drawing quality traffic to the Internet

Intervention. In 2006, the CDC published a case study of their Google and Yahoo marketing campaign for the CDC's breast cancer awareness site. The campaign was timed to coincide with the National Breast Cancer Awareness Month during the month of October. Their methods included keyword development and Ad group development (see below for specifics). Results from their campaign's were successful. Via Google Adwords, they garnered 152,709 ad impressions over 6 weeks time. Those impressions garnered 8,407 clicks for a click through rate of 5.5%. During the same time their Organic Search click through was 0.3%. Although, it is important to note that particular keywords performed significantly better than others – one in particular garnering a 14.6% click through rate. Comparing the 6 weeks of the campaign to the 6 weeks after, Total Page Visits for all target pages was 43,837 compared to 10,301 post-campaign.

CDC Methods:
Keyword development: As discussed in Section 1.1, keyword lists that were initially developed for the metatag data of the site were the starting point for the development of keywords for the ad campaign. Table 1 shows tools used by the CDC to refine and expand initial keyword list.

Table 1: Tools for developing Keyword Lists

Tool 1:	Reviewed keyword terms used for the current National Breast and Cervical Cancer Early Detection Program Web site.
Tool 2:	Reviewed CDC Search Terms (Top 1000 queries) for relevant terms.
Tool 3:	Reviewed the results of the analysis of CDC breast cancer search terms for August 2005, as provided by Jeanie Barker.
Tool 4:	Used Google AdWords Keyword Tool to identify useful terms relevant to breast cancer and mammograms (Keyword Variations).
Tool 5:	Used Google AdWords Keyword Tool to identify terms currently used by other organization Web sites such as the American Cancer Society (Site-Related Key).
Tool 6:	Used Ask.com Sponsored Listings Keyword Tool to identify useful terms relevant to breast cancer and mammograms.

Source: Center for Disease Control and Prevention, 2007

- Ad Group Development: Ads and Keywords were topically grouped into Ad Groups. An Ad Group has a single destination, but multiple ad groups could utilize the same destination with overlapping keywords. The CDC's Breast Cancer Awareness campaign ended up with 9 Ad Groups, targeting 5 destination URL's, with 29 Ads and 135 keywords.

2.2 <u>Other Types of Online Advertising</u>

- Social Networking Sites: While the benefits of communication via Social Networking sites such as Facebook, MySpace and Twitter will be covered more fully in Section 3.0, one option is to purchase ad space that can be highly targeted based on a user's location or interest. For example, on Facebook, it is fairly simple to set up an ad campaign that is demographically targeted, with daily budget limits.

- Banner Ads: Banner ads can be placed on any site that allows it. This includes health information sites such as WebMD. It is less targeted but can be effective during a campaign.

3.0 Social Media:

Over the past few years, participation in Social Networking Sites, as well as other forms of Social Media, such as blogs and forums, has increased dramatically (Center for Disease Control and Prevention, 2009; Pew Internet & American Life Project, 2009, 2010). Again, in 2009 the CDC released Best Practices for using Social Media to disseminate quality web-based Health Information. Within this report Social Media is described as an effective way to "expand reach, foster engagement, and increase access to credible, science-based health messages."

However, the use of Social Media as a dissemination tool can be costly in terms of staff time resources. This report makes it clear that within the Social Media realm, as you move from Dissemination to Engagement the Overall Cost in terms of Staff Time and Money moves from low to high. Examples of low cost tools are Buttons/Badges, Widgets and e-Cards. Higher cost examples are Blogs, Texting and Social Networks.

Table 2 describes the CDC's top lessons learned in their dissemination efforts via Social Media.

Table 2: CDC's Top Social Media Lessons Learned

**Lesson 1**	**Make Strategic Choices and Understand the Level of Effort**<br>Be strategic and follow demographic and user data to make choices based on audience, communications objectives, and key messages. Be sure to assess the level of effort needed to maintain these channels and ensure you have the necessary time and effort to commit to your efforts. Often, the resources needed to start and maintain social media projects are different than traditional communication efforts.
**Lesson 2**	**Go Where the People Are**<br>Social media can help reach people where they are—millions of people use social media and spend a lot of time in these spaces learning, sharing, and interacting. The popularity of key social media sites can be assessed by reviewing user statistics and demographics. Additionally, there are several niche social networking sites that target specific groups, like moms, physicians, or racial and ethnic groups, or sites that focus on a particular topic like travel or health.
**Lesson 3**	**Adopt Low-Risk Tools First**<br>If you are starting out and finding resistance to using social media among your communication team or stakeholders, it may be helpful to first adopt low▯risk solutions and later build on your successes. Products such as podcasts, videos, and widgets are easily downloadable, and can be accessed from partner sites and posted on your website.
**Lesson 4**	**Make Sure Messages are Science-Based**

	As with any effective health communication, messages developed for dissemination through social media channels should be accurate, consistent, and science‐based.
**Lesson 5**	**Create Portable Content**<br>Develop portable content—such as widgets and online videos—that can easily extend reach beyond your website to provide credible, timely, and accurate content for partners and others who want to help spread your health messages.
**Lesson 6**	**Facilitate Viral Information Sharing**<br>Make it easy for people to share your messages and become health advocates. This can be accomplished by using social media sites such as Facebook and YouTube that encourage sharing among users, or you can use tools with sharing features, like widgets or eCards.
**Lesson 7**	**Encourage Participation**<br>Social media allows for the tailoring of messages to help express empathy and acknowledge concern, promote action, and listen to what people are saying about health‐related topics in your community. Two‐way conversations can foster meaningful communication with your audiences that can help to facilitate relationships, sharing, and interaction.
**Lesson 8**	**Leverage Networks**<br>Social media allows people to easily establish networks that they can access on a regular basis. For example, Facebook reports the average Facebook user has 130 friends, or a network of 130 people with whom they can easily share information, and may choose to share your health messages (Facebook. 2010). By strategically leveraging these established networks you can facilitate information sharing and in turn, expand the reach of your message.
**Lesson 9**	**Provide Multiple Formats**<br>Providing messages in multiple formats increases accessibility, reinforces messages, and gives people different ways to interact with your content based on their level of engagement and access to media.
**Lesson 10**	**Consider Mobile Phones**<br>Over ninety percent of adults in America subscribe to mobile services. Therefore, mobile technologies such as text messaging and mobile websites offer an opportunity to rapidly reach a large percentage of your audience members no matter where they are.
**Lesson 11**	**Set Realistic Goals**<br>Social media can raise awareness, increase a user's knowledge of an issue, change attitudes, and prompt behavior change in dynamic, personalized, and participatory ways. However, like traditional communication, social media alone may not be able to meet all of your communication goals or address all of the target audiences' needs. Set your goals accordingly.
**Lesson 12**	**Learn from Metrics and Evaluate Your Efforts**<br>Digital communications offer many metrics that you can use to focus and improve your communications efforts. Metrics can help you to report usage, monitor trends, and gauge the success of specific promotions or

	outreach efforts. Beyond simple metrics, social media efforts can also be evaluated by measuring the use of information, engagement, and participation of people with your content, and its health impact. Monitoring trends and discussions on social media networks can also be a valuable way to better understand current interest, knowledge levels, and potential misunderstandings or myths about your health topic.

Source: Center for Disease Control and Prevention, 2009

4.0 Things to Consider in Developing a Dissemination Campaign: An Internet-based Intervention Promoting Safe Behaviors Among Young Drivers

- Target Your Audience
  - I. Teenagers – Age 15-18, Learning to Drive or Newly Licensed
  - II. Parents of Teenagers who are either learning to drive or newly licensed
  - III. Educators of Teenagers who are either learning to drive or newly licensed
- Determine Your Objective
  - I. Encourage Safe Driving Behaviors
  - II. Reduce the incident of MVC's involving Teen Drivers and related deaths
  - III. Increase the use of Web-Based Intervention aimed at Encouraging Safe Behaviors amongst Teen Drivers and Passengers
- Describe Audience Communication Needs
  - I. Teenagers: Avid Internet Users, moving more towards mobile devices, texting, social network sites
  - II. Parents: Avid Internet Users, also moving towards mobile devices but not as quickly as teens, email users, social network sites
  - III. Educators: Becoming more Web-savvy, Increasingly using Web to facility Educational experience
- Goal Integration
  - I. Research to Action
  - II. Coordinate with National Teen Driver Safety Week
- Message Development
  - I. Teens: "On-line Drivers Ed"
  - II. Parents: "Teach Your Teen How to Drive Safely"
  - III. Educators: "Effective On-line Drivers Ed Program"
- Organizational Resources and Capacity
  - I. Determine who will be responsible for implementation of dissemination campaign and the number of hours that can be allocated for content creation and maintenance.
- Identify Social Media Tools
  - I. Teens: Facebook, MySpace, Mobile Devices
  - II. Parents: Facebook, Internet Search Engines
  - III. Educators: Facebook, Internet Search Engines
- Define Activities
  - I. Develop Facebook and MySpace page dedicated to Teen Driving Safe Behaviors
  - II. Search Engine Optimize Intervention
  - III. Introduce Adwords Campaign during National Teen Drivers Safety Week

IV. Introduce Facebook and MySpace advertising campaign targeting Teens and Parents of Teens, and Educators of Teens

- Identify Key Partners
  I. Injury Prevention Programs
  II. School Districts
  III. Drivers Ed Training Programs
  IV. Parents Groups – Parent / Teacher Organizations
- Define Measures of Success for Evaluation
  I. Number of Teens and Parents accessing Web-based Intervention
  II. Decrease in MVC's involving Teen Drivers
  III. Decrease in Mortality Rate related to MVC's involving Teen Drivers
- Evaluate
  I. Create Evaluation Plan, See Report #7 for more information.

5.0 Resources

- Search Engine Optimization for the Layperson, Imaginary Landscapes, http://dev.imagescape.com/library/whitepapers/seo.pdf.
- Marketing Campaign on Google, Yahoo!, and WebMD for the CDC Breast Cancer Website 2006, CDC, http://www.cdc.gov/healthmarketing/pdf/Web_site_marketing_breast_cancer_2006_Final_Report_andCover_rev2.pdf.
- The Health Communicator's Social Media Toolkit, CDC, 2009, http://www.cdc.gov/healthcommunication/ToolsTemplates/SocialMediaToolkit_BM.pdf

6.0 References

4.3.11 Technical Report: Evaluation framework to assess use patterns for Internet-based interventions

Overview

This report aims to describe the use of Web Analytics to evaluate use patterns of an Internet-based Intervention. Effective evaluation should result in the development of Key Performance Indicators (Jansen 2009). Key performance indicators (KPIs) "measure performance based on articulated goals for the business, user understanding, or Web system" (Jansen 2009). For the purposes of evaluating use patterns of web-based interventions, Key Performance Indicators (KPI) should be defined as Participant Exposure, or Webpage viewing, as well as Participant Engagement, or Viewing Duration (Danaher and Seeley 2009).

An efficient but imperfect way of getting at these KPI's of an Internet-based intervention is through Web Analytics or Site Analytics. Web Analytics is defined by the Web Analytics Association as "the measurement, collection, analysis, and reporting of Internet data for the purposes of understanding and optimizing Web usage" (Web Analytics Association). It is

important to note that although Web Analytics can be an extremely valuable tool, the science is imperfect with error rates in the 5-10% range (Jansen 2009).

That said, utilizing Web Analytics to evaluate the reach of dissemination efforts can be invaluable in helping determine our two defined KPI's – Participant Exposure and Participant Engagement – by allowing the study of a participant's interaction with an Internet-based intervention (Jansen 2009).

1.0 Metrics

In relation to Web Analytics, metrics are the different kind of measures available for analyzing user information. According to Jansen et al there are 8 common metrics used in analyzing website usage:

Metric	Description
1. Demographics and System Statistics	The physical location and information of the system used to access the Website
2. Errors	Any errors that occur while attempting to retrieve page
3. Internal Search Information	Information on keywords and results pages viewed using a search engine embedded in the Website
4. Referring URL and Keyword Analysis	Which sites have directed traffic to the Website and which keywords visitors are using to find the Website
5. Top Pages	The pages that receive the most traffic
6. Visit Length	The total amount of time a visitor spends on the Website
7. Visitor Path	The route a visitor uses to navigate through the Website
8. Visitor Type	Who is accessing the Website – returning, unique, new

Source: Jansen 2009

2.0 Evaluation of Participant Exposure

Referring to the table above, metrics of interest that help us get at Participant Exposure and the measures utilized to analyze it are:

Metric	Measures via Google Analytics
I. Demographics and System Statistics	City, Country/Region, Languages, Browser Capabilities, Network Properties, Mobile Devices used
II. Internal Site Search	Overall Usage, Search Terms used, Start Pages, Destination

	Pages
III. Referring URL and Keyword Analysis	Traffic Sources: Direct, Referring, Search Engines; Keywords Used; Advertising: Keywords used, Campaigns, Ads, Adgroups
IV. Top Pages	Content: Top Content, Top Landing Pages, Top Exit Pages, Event Tracking
V. Visitor Type	New Visitors, Unique Visitors, Return Visitors

3.0 Evaluation of Participant Engagement

Metrics of interest that help us get at Participant Engagement are:

Metric	Measures via Google Analytics
I. Visit Length	Length of Visit, Depth of Visit, Time on Site, Bounce Rate
II. Visit Path	Pageviews, Average Pageviews,

Combining measures of Participant Exposure - such as traffic sources or referring sources - with measures of Engagement - such as Average Time on Site and Bounce Rate – we gain a better understanding of the quality of traffic to the site and which techniques are producing a higher quality of traffic. For example, below is a screenshot of Top Keywords directing traffic to the site. By also reviewing 1) Pages/Visit; 2) Avg Time on Site; and 3) Bounce Rate, you get a sense of the relationship between the source keyword and participant engagement.

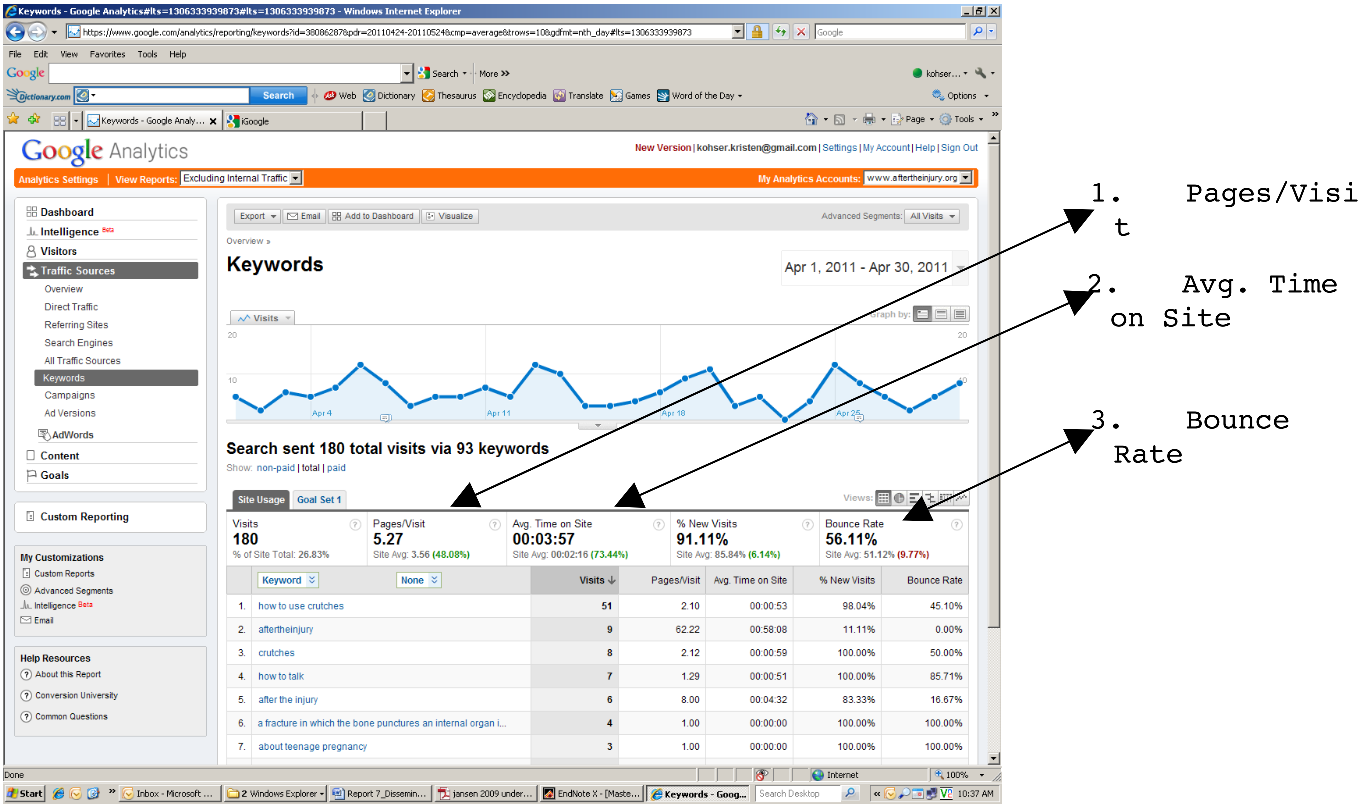

4.0 Resources

<u>4.1 Use of Web Analytics to Evaluate an Online Marketing Campaign</u>:
Marketing Campaign on Google, Yahoo!, and WebMD for the CDC Breast Cancer Website 2006, CDC, 2007, www.cdc.gov/healthmarketing/pdf/Web_site_marketing_breast_cancer_2006_Final_Report_andCover_rev2.pdf.

<u>4.2 Use of Web Analytics to Evaluate Various Social Media Tools</u>:
The Health Communicator's Social Media Toolkit, CDC, 2009, www.cdc.gov/healthcommunication/ToolsTemplates/SocialMediaToolkit_BM.pdf

5.0 References

Center for Disease Control and Prevention (2007). Marketing Campaign on Google, Yahoo!, and WebMD for the CDC Breast Cancer Web Site – 2006.

Center for Disease Control and Prevention (2009). The Health Communicator's Social Media Toolkit.

Danaher, B. and J. Seeley (2009). "Methodological Issues in Research on Web-Based Behavioral Interventions." <u>Annals of Behavioral Medicine</u> **38**(1): 28-39.

Jansen, B., Ed. (2009). <u>Understanding user-web interactions via web analytics</u>. Synthesis Lectures on Information Concepts, Retrieval, and Services.

Maloney, B. (2005). Search Engine Optimization for the Layperson, Imaginary Landscape**:** 4.

Pew Internet & American Life Project. (2010). "Email vs. Social Networks." Retrieved May 20, 2011, from http://pewresearch.org/databank/dailynumber/?NumberID=1088.
Web Analytics Association. Retrieved May 20, 2011, from http://www.webanalyticsassociation.org/?page=aboutus.

4.4 Accomplishments Report for Year 5 Milestones: July 1, 2013 – December 31, 2013

4.4.1 Milestones for Year 5 (July 1, 2013 – December 31, 2013)

**Milestones**	**Project Aims Satisfied**	**Annual Report Submission**
1. Analyze the results of the marketing/dissemination study	2	7/1/2013-12/31/2013
2. Complete work for all methods	1, 2	7/1/2013-12/31/2013
3. Complete final report	1, 2	7/1/2013-12/31/2013
4. Prepare presentations and papers for publication	1, 2	7/1/2013-12/31/2013

4.4.2 Summary of the analysis of the results of the marketing/dissemination study (Year 5, Milestone 1)

Purpose
Little is known about the use of Twitter chats to effectively disseminate health information and about how well health-specific content is transmitted within Twitter networks. Methodological techniques involved in evaluating data from Twitter chats for research purposes have been limited. Here we present an evaluation of two objectives: 1) the use of a Twitter chat to raise awareness about teen driver safety and 2) a pilot analysis of Twitter tweet and re-tweet content to better understand how information is disseminated within a novel Twitter re-tweeting network on child and teen injury.

Methods - Twitter chat

The Center for Injury Research and Prevention (CIRP) facilitated an hour-long Twitter chat during National Teen Driver Safety Week on October 18, 2012 under #teendriving2012. Key stakeholders including State Farm, the National Highway Traffic Safety Administration, CDC, AAA, physicians, researchers, and community members were invited to participate. Topics for discussion were developed by CIRP and multiple references to teendriversource.org, a CIRP-maintained website geared towards teen driver safety, were imbedded into tweet content. A combination of Google Analytics and the Twitter

Application Programming Interface (API) were used to extract and analyze quantitative data regarding the chat. The following metrics were collected:

- Total # of tweets from all unique users in Twitter chat
- Following base of all unique users in the chat including the average # of tweets they send
- Total # of re-tweets generated from the chat
- Proportion of unique users that re-tweet @safetyMD
- Unique visitors driven to teendriversource.org before, during, and after the Twitter chat

Methods – Twitter tweet and re-tweet content

During a four month time period (July-October 2012), the Children's Hospital of Philadelphia's Center for Injury Research and Prevention's (CIRP) Twitter handle, @safetyMD, was monitored for content. Content on tweet and re-tweeting patterns were observed and recorded. We defined our "Layer 0" as the tweets by @safetymd. "Layer 1 Re-tweeting Network" as those who re-tweeted content from @safetymd; our "Layer 2 Re-tweeting Network" as those who re-tweeted content from the Layer 1 Re-tweeting Network. The Twitter Application Programming Interface (API) was used to extract tweet and re-tweet content from all layers including unique handles, date/time of tweets/re-tweets and the text content. An exploratory round of content analysis was conducted by calculating frequencies of the top re-tweeted words.

Results – Twitter chat

A total of 435 tweets were generated from 150 unique Twitter users. These 150 users had a cumulative following base of 658,120 (mean=4,387) and a cumulative history of composing 864,698 tweets (mean=5,765). A total of 137 re-tweets were generated and 10% of the unique users re-tweeted the CIRP facilitator 25 times. This group had a cumulative following base of 61,143 (mean=4,076) and generated a total of 58,796 (mean=3920) historical tweets. Compared to the average number of unique visitors per day for the entire month of October 2012, there was a 41% increase on the day of the Twitter chat.

Results - Twitter tweet and re-tweet content

A total of 6,113 words were tweeted in layer 0. The top words in this layer included teens, driving/drive, safety/safe, and injury and cumulatively represented about 10% of all words tweeted. Within layers 1 and 2, a total of 3,711 and 60,795 words were re-tweeted respectively. The percentage of key words in these two layers dropped to 6% in layer 1 and 2.5% in layer 2.

Overall conclusions from both objectives

Objective 1: Our results may suggest that a Twitter chat can be a useful technique to disseminate health information. The data presented above may be proximal indicators of greater inclusion, message dissemination and grassroots action. Increases in traffic to our website may indicate that a Twitter chat engaged participants, because they clicked on the links to resources. Twitter can be an important tool in a health communication tool chest, but evaluation needs to catch up with the technology to determine its effectiveness for health promotion. Twitter is free, but using it effectively requires staff resources and a degree of faith. We will continue to monitor our Twitter metrics and general E-Health trends to guide future decisions.

Objective 2: While there was strong initial dissemination of messages from @safetyMD, our preliminary results using the methods developed for this grant indicate a strong dilution effect through the social media network (also known as the "Twitterverse"). Our @safetymd dissemination strategy aimed to utilize the viral nature of Twitter to spread evidence-based injury prevention information. While there was evidence of dissemination, only approximately 2.5% of the content reached a second stage of spread. Further strategies are needed to facilitate more efficient dissemination of health information through Twitter.

This line of research, funded by the Pennsylvania Department of Health, demonstrated the importance of rigorous and novel evaluation methods to ensure that the communication/dissemination strategies are effective. In our analysis, Twitter had very limited effectiveness in broad dissemination of safety messages and this might also be the case with other Twitter-based health information dissemination strategies. Additional larger studies are needed to corroborate these findings.

Our results were presented as two poster presentations at the 2013 International Society for Research on Internet Interventions (ISRII) in Chicago, IL and an oral presentation at the 2013 Medicine 2.0 conference in London, UK. We have also submitted a manuscript describing the quantitative techniques used to differentiate clusters of a sample Twitter users following @safetyMD based on their tweet content.

4.4.2 Prepare presentations and papers for publication (Year 5, Milestone 4)

In addition to the previously published studies, we are currently in the process of preparing additional manuscripts from our results from Aim 1 and Aim 2. Section 20 of the main document outlines the manuscripts that have been submitted to date.

References (Does not include references for embedded reports)

1. NHTSA. *Fatality Analysis Reporting System Encyclopedia*. [cited March 2, 2008]; Available from: http://www-fars.nhtsa.dot.gov/Main/index.aspx

2. Committee on Trauma Research, et al., *Injury in American: A Continuing Public Health Problem*. 1985, Washington, DC: National Academy Press.

3. Committee to Review the Status and Progress of the Injury Control Program at the Centers for Disease Control, *Injury Control: A Review of the Status and Progress of the Injury Control Program at the Centers for Disease Control*. 1988, Washington, DC: National Academy Press.

4. Institute of Medicine, Reducing the Burden of Injury: Advancing Prevention and Treatment. 1999, Washington, DC: National Academy Press.

5. Graham, R., and Gootman, J.A. *Preventing teen motor crashes: contributions from the behavioral and social sciences and summary of the report of the National Research Council and Institute of Medicine.* American Journal of Preventive Medicine 35.3 (2008): S253-S257.

6. Backer, T.E., E.M. Rogers, and P. Sopory, *Designing health communication campaigns: What works?* 1992, Newbury Park, CA: Sage.

7. McQuail, D., Communication. 1987, New York: Longman Press.

8. Rogers, E.M. and J.D. Storey, *Communication campaigns, in Handbook of communication science*, C.R. Berger and S.H. Chaffee, Editors. 1987, Sage: Newbury Park, CA.

9. Fox, S. and S. Jones, The Social Life of Health Information: Americans' pursuit of health takes place within a widening network of both online and offline sources. 2009, Pew Internet & American Life Project: Washington, DC.

10. Fishbein, M., The role of theory in HIV prevention. AIDS Care, 2000. 12: p. 273-278.

11. Bennett, G.G. and R.E. Glasgow, The delivery of public health interventions via the Internet: actualizing their potential. Annual Review of Public Health, 2009. 30: p. 273-292.

12. Kelman, H.C., Processes of opinion change. Public Opinion Quarterly, 1961. 25: p. 57-78.

13. Gentry, E.M. and C.M. Jorgensen, Monitoring the exposure of “America Responds to AIDS” PSA campaign. Public Health Reports, 1991. 106: p. 651-655.

14. Gillispie, M.A. and L.B.M. Ellis, Computer-based patient education revisited. Journal of Medical Systems, 1993. 17(3/4): p. 119-125.

15. Dorn, L. and D. Barker, The effect of driver training on simulated driving Performance. Accident Analysis and Prevention, 2005. 37: p. 63-69.

16. Garay-Vega, L., F. D.L., and A. Pollatsek, Hazard Anticipation of Novice and Experienced Drivers: Empirical Evaluation on a Driving Simulator in Daytime and Nighttime Conditions. Transportation Research Record, 2007. 2009: p. 1-7.

17. Winter, J.C.F., et al., Relationships between driving simulator performance and driving test results. Ergonomics, 2009. 52(2): p. 137-153.

18. Bernhardt, J.M., Improving Health Through Health Marketing. Preventing Chronic Disease: Public Health Research, Practice & Policy, July 2006. 3(3): p. 1-3.

19. Grier, S. and C.A. Bryant, Social Marketing in Public Health. Annual Review of Public Health, 2005. 26: p. 319-339.

20. McDonald, C., C, Tanenbaum, J.B., Lee, Y.-C., Fisher, D.L., et al., "Using Crash Data to Develop Simulator Scenarios for Assessing Young Novice Driver Performance " Transportation Research Record: Journal of the Transportation Research Board 2321: 73-78, 2012.

21. Winston, F.K., McDonald, C.C., Kandadai, V., Loeb, H., et al., "Headway Time Errors, Safe Driving Skill, and Experience: An Initial Validation of the Simulated Driving Assessment," Transportation Research Board. 2014. Paper 14-4867.

22. McDonald, C.C., Seacrist, T., Lee, Y.C., Loeb, H., et al., "Headway Time and Crashes Among Novice Teens and Experienced Adult Drivers in a Simulated Lead Truck Braking Scenario," 7th International Driving Symposium on Human Factors in Driving Assessment, Training, and Vehicle Design. 2013. 439-445.

23. Winston, F.K., McDonald, C.M., Kandadai, V., Seacrist, T., &Winston, Z. “Experience and skill predict failure to brake errors: Further validation of the simulated driving assessment,” SAE 2014 World Congress & Exhibition Compendium of Papers. (In Press).

Appendix

A.1.

PROTOCOL SYNOPSIS

(TEMPLATE & EXEMPLAR PROTOCOL)

**Study Title**	**Web-based Intervention for Safe Teen Driving: Content Pre-testing**
**Funder**	
**Study Rationale**	As the use of Web-based Health Interventions grows, so does the need to ensure that these are quality interventions. Thus the need for systematic, thorough and scientifically rigorous evaluation and testing. This protocol sets forth a framework for a formative evaluation to pretest content and prototype of a web-based intervention aimed at improving teen driving.
**Study Objective(s)**	**Primary**<br>The primary objective is to pretest the content and prototype of the web-based intervention aimed at reducing the morbidity and mortality associated with MVC's involving teen drivers.<br>**Secondary**<br>• To evaluate feasibility and usability of the intervention, and<br>• To inform any necessary modifications of the intervention for future Efficacy and Effectiveness evaluations.
**Study Design**	Evaluation of web-based intervention utilizing qualitative and quantitative feedback, via focus groups, interviews, satisfaction questionnaires, and task analysis, from teenagers and parents using the web-based intervention modules and what they learned from the materials.
**Subject Population key criteria for Inclusion and Exclusion:**	**Inclusion Criteria**<br>1. Subjects age 15 - 18<br>2. Teenager newly licensed within past 6 months<br>3. One parent or legal guardian willing to participate<br>4. Speaks English well enough to complete web-based intervention, complete questionnaires and participate in interview<br>**Exclusion Criteria**<br>1. Obtained Driver's License post 6 months<br>2. No parent or guardian available
**Number Of Subjects**	60 children ages 15-18 and one parent/guardian. To be enrolled at CHOP through referral via enrollment in the Philadelphia School District's Driver's Training Program.
**Study Duration**	The study duration per subject will be one visit of approximately one hour.<br>The entire study is expected to last no longer than six months.
**Study Phases**	**Screening:** Demographic and stage of learning how to drive information to ensure

**Screening**<br>**Study Treatment**<br>**Follow-Up**	eligibility.<br>**Visit 1**: Subjects will be led through specific task using the web-based intervention and asked to complete. Feedback will be gathered after each task is completed. Feedback will be recorded via screen capture and/or audio- or video-recording. Subjects will then complete a Satisfaction Questionnaire to gather their overall impressions and suggestions.
**Efficacy Evaluations**	Primary evaluation criteria:<br>Qualitative description of subjects' reactions to web-based teen driving intervention.
**Safety Evaluations**	Not applicable. (Primarily informational / educational intervention, with very low risk for upset or distress. Research staff will monitor and document subjects' responses, and offer referrals for assistance if needed.)
**Statistical And Analytic Plan**	Descriptive analyses of subject reactions and ratings.
**Data And Safety Monitoring Plan**	The PI will serve as data and safety monitor.
